# A data-driven framework for modeling the dendritic spine continuum using dimensionality reduction and clustering toward understanding synaptic plasticity

**DOI:** 10.1371/journal.pone.0349775

**Published:** 2026-06-02

**Authors:** Uma Shashi Sharma, Philip R. LeDuc, Yongjie Jessica Zhang

**Affiliations:** Department of Mechanical Engineering, Carnegie Mellon University, Pittsburgh, Pennsylvania, United States of America; Harvard University, UNITED STATES OF AMERICA

## Abstract

Dendritic spines are dynamic extensions of dendrites that change in shape and distribution in response to neuronal activity, playing central roles in memory and learning. Computational methods are widely used to characterize spine morphology, yet feature selection, dimensionality reduction, and clustering choices are often made *a priori* and evaluated independently, and as a result it remains unclear how analysis decisions influence low-dimensional representations of spine shape and the biological interpretations drawn from them. We present a decision-based visual characterization framework that systematically evaluates dimensionality reduction and probabilistic clustering strategies for dendritic spine morphometry. Using a labeled two-photon laser scanning microscopy (2PLSM) dataset and a secondary dataset with differing imaging conditions to assess generalization, we compare PCA, ISOMAP, t-SNE, UMAP, and PCUMAP alongside hierarchical clustering, Fuzzy C-Means, and Gaussian Mixture Models. We additionally introduce a Biological Transition Score (BTS) to quantify how well low-dimensional embeddings reflect known developmental and functional relationships among spine types. Across datasets, dimensionality reduction methods capture complementary aspects of spine morphology. On the primary dataset, nonlinear approaches better preserve fine-scale structure, with PCUMAP providing a favorable balance between local structure preservation and global continuity. In contrast, analysis of a lower-resolution secondary dataset shows that PCA is more robust under increased feature-level noise. These findings demonstrate that the optimal dimensionality reduction strategy is dataset-dependent, underscoring the importance of systematic, data-driven method selection. When paired with probabilistic clustering, these representations reveal a morphological continuum that bridges classical “mushroom,” “stubby,” and “thin” spine categories. Increasing the number of identified sub-groups preserves or strengthens structural organization relative to expert-labeled classes, demonstrating that weakly supervised representations can resolve intra-class heterogeneity beyond discrete manual classifications. This framework provides a structured, quantitative approach for selecting dimensionality reduction and clustering strategies, enabling more consistent and biologically grounded interpretations of dendritic spine morphology.

## Introduction

The ability of the brain to receive, process, and transmit information relies on dynamic connections between neurons. Dendritic spines, the microscopic protrusions along the branched extensions of nerve cells, are essential to this process, serving as key sites for receiving synaptic information by forming functional connections with neighboring neurons’ axons [1,2]. These tiny structures, ranging from only 0.2–2μm in length [3], exhibit significant morphological diversity and plasticity, constantly changing shape in response to neural activity. Furthermore, the complexity, shape distributions and density of dendritic spines are associated with the strength and function of synaptic connections, indicative of calcium dynamics, receptor location, and in turn, the probability of postsynaptic firing [1]. Thus, modeling dendritic spines has tremendous promise for progressing our understanding of brain development and cognitive flexibility [2].

Furthermore, deficits in spine shape and density have been observed in various cognitive disorders and intellectual disabilities, including Traumatic Brain Injury (TBI), Schizophrenia, Alzheimer’s Disease, Down Syndrome, Rett Syndrome, and even chronic stress and anxiety [4–6]. For example, individuals with Down Syndrome, a genetic condition resulting in intellectual disability, present with significant reductions in dendritic spine length and density [4]. Because these smaller-scale structural changes appear long before visible large-scale brain damage, dendritic spines serve as early markers of disease, and studying them can reveal how these conditions progress, enabling better diagnosis and treatment.

Traditional dendritic spine characterization methods classify spines into three to five discrete groups (“mushroom”, “thin”, “stubby”, and sometimes “branched” and “filopodia”) based on morphological geometries [2,7]. These geometries are associated with function: mushroom spines have large heads and small necks, and are associated with strong, long-term synaptic connections (lasting weeks to months). Thin and stubby spines are more dynamic; thin spines have small heads with long necks and are involved in synaptic plasticity. Stubby spines lack a distinct neck and are common in early development, both potentially transitioning into mushroom spines over time [1,2]. However, multiple reports have challenged traditional classification methods: As spines develop, they transition from one shape category to another, existing on a continuum rather than in discrete categories [8–12]. Furthermore, the classification approach for dendritic spine analysis may obscure relevant biological features of the data, resulting in a loss of information about feature correlations and spine shape variations [11,13].

Computational approaches, including classification and clustering, have great promise for generating accurate and reproducible models of dendritic spine shape to improve our understanding of neuronal mechanisms of information transfer [14]. Early efforts to group dendritic spines based on shape characteristics [15] revealed morphological subgroups within the “mushroom” class, highlighting heterogeneity within traditional spine categories. Subsequent work introduced probabilistic models to capture transitions between spine shapes, providing initial evidence that spine morphology is better described as a continuum rather than as discrete classes [10]. Since then, multiple studies have expanded on these foundational methodologies using improved segmentation algorithms [16–19], higher image resolution [20,21], alternative computational methods [21,22], and visualization methods [18,23,24] for both classification and clustering of dendritic spine shape. However, most existing studies either examine dimensionality reduction or clustering in isolation, or adopt a single representation and clustering strategy *a priori*, without systematically evaluating how alternative choices influence the resulting organization of spine morphology. Linear dimensionality reduction, commonly principal component analysis (PCA), is frequently used to represent high-dimensional morphometric features in a lower-dimensional visual space [10,18,21], even though PCA is limited in its ability to capture nonlinear structure and complex shape relationships. Additionally, many approaches still impose discrete categories, which may obscure gradual transitions and continuous variation in spine shape [2,21,25]. Building on our group’s prior work in neuroanatomical modeling and computational morphology [17,26–30], we address this gap by systematically comparing multiple dimensionality reduction and clustering strategies to identify representations that best enable visualization of morphological relationships. This approach to modeling the dendritic spine continuum strives for improved representation of spine morphological transitions and subtypes within traditional morphological classes.

This study presents a weakly supervised, feature-driven decision framework for visualizing dendritic spine morphometry and systematically evaluating continuous representations of spine shape variation. Applying this decision framework reveals the continuous landscape of dendritic spine morphology, with gradual transitions between classical spine categories rather than sharply separated classes, demonstrating how representation selection influences the observed organization of spine shape.

The key contributions of this work include: (1) A comprehensive, multi-feature representation of dendritic spine morphology. We extract 35 quantitative features capturing shape, contour, and intensity information from two-photon laser scanning microscopy (2PLSM) images, providing a holistic description of dendritic spine structure that expands beyond prior approaches relying on limited, manually selected geometric descriptors. (2) A systematic, decision-based comparison of dimensionality reduction strategies. We evaluate both linear (PCA) and nonlinear (ISOMAP, t-SNE, UMAP, and PCUMAP) dimensionality reduction methods within a unified framework, quantifying how different embeddings preserve structural organization in high-dimensional morphometric data, providing a practical guidance for selecting representations based on objectives such as local continuity, global structure, and interpretability. (3) Weakly supervised, probabilistic modeling of spine morphology without predefined classes. Rather than enforcing rigid categories, we apply probabilistic clustering to low-dimensional embeddings to uncover natural groupings and continuous variation in spine morphology to allow transitional spine states to emerge directly from the data. (4) Biologically interpretable shape transitions. By modeling spine morphology as overlapping probabilistic groupings rather than discrete classes, our framework enables visualization and quantification of intermediate forms that may reflect functional, developmental, or activity-dependent remodeling, supporting a more nuanced biological interpretation than static classification approaches.

## Methods

### Quantification of dendritic spine shape

To quantify dendritic spine morphology, we collected a set of shape, contour, and intensity-based characteristics from two-photon laser scanning microscopy (2PLSM) images of dendritic spines [31,32]. The correlation between dendritic spine shape and function has been extensively researched [1,9,33,34], and the features collected were motivated by multiple reports that point to shape, contour, and intensity features of 2PLSM images being indicative of dendritic spine morphology and as a proxy for describing function [10,15,21,35]. Shape-based morphological features, such as spine neck length, neck width, and head diameter, were collected to provide insights into signal compartmentalization and synaptic strength [35]. Contour-based features, such as convex hull ratio, circularity, and eccentricity, were measured to convey subtle irregularities, areas of high complexity, or elongation of the spine, providing additional insight into geometry [21]. Intensity features based on histograms of gradients (HOG) and Gray Level Co-Occurrence matrices (GLCM) were measured to discriminate spines with thinner necks and highlight intensity differences between the spine neck and head, which could be indicative of signal compartmentalization or synaptic strength [31]. In this manuscript, we use a 2PLSM hippocampal dendritic spine dataset from Ghani et al. as the primary dataset to demonstrate our computational and decision framework. We perform an additional analysis on a secondary dataset from Smirnov et al., which also contains 2PLSM-imaged hippocampal dendritic spine images but differs in image acquisition protocol, demonstrating the generalization of this framework across imaging conditions. Details of the two open-source datasets and our feature collection pipeline are detailed in the Datasets and Data Preparation section.

### Dimensionality reduction

#### Dimensionality reduction techniques.

We explored multiple dimensionality reduction methods to transform each spine’s high-dimensional feature set into a more interpretable, low-dimensional representation that retained the intrinsic relationships between features in the original high-dimensional space. While most existing computational pipelines for dendritic spine analysis use PCA for dimensionality reduction [10,18,21], we hypothesized that non-linear dimensionality reduction methods may better represent the intrinsic structure of the data in low-dimensional space.

We applied five dimensionality reduction methods to the dataset: PCA, Isometric Mapping (ISOMAP), t-Distributed Stochastic Neighbor Embedding (t-SNE), Uniform Manifold Approximation and Projection (UMAP), and Pearson’s Correlation Uniform Manifold Approximation and Projection (PCUMAP). The first four methods were chosen to represent both linear and nonlinear approaches used in dendritic spine and neuroscience research for analyzing complex morphological data. Finally, PCUMAP is a state-of-the-art dimensionality reduction technique that aims to overcome some of the limitations of other methods in representing both local and global structure.

Table 1 summarizes the key components of five dimensionality reduction techniques that were chosen. Each of these techniques has strengths and limitations related to sensitivity and ability to preserve local and global structure. Global linear techniques like PCA are popular for their interpretability, but have their limitations: since PCA assumes linearity, the method cannot capture underlying nonlinear structure in the data [10,36]. Manifold approaches like ISOMAP attempt to solve the issue of nonlinearity by using local Euclidean distances from the original feature space in the low-dimensional space, but in recent years these techniques have been shown to misrepresent neighborhood structure [36], [25]. t-SNE and UMAP are other nonlinear methods that are able to capture local structure, meaning they are effective at creating separable classes and clusters. Additionally, given that these methods are nonlinear, they may be better able to model the continuous nature of spine shape growth [23]. However, both of these methods have issues preserving global structure [36]. For this reason, we also apply a recently developed method, PCUMAP, which aims to both preserve local and global structure using correlation-based loss [37]. Given that all linear and nonlinear methods have pros and cons for our purposes, we decided to test all five using a comprehensive set of local and global feature preservation metrics. The hyperparameters used for each dimensionality reduction method are detailed in S4 Table in S1 File.

**Table 1:** Comparison of Dimensionality Reduction Methods for Dendritic Spine Analysis.

Method	Type	Approach	Benefits	Limitations
PCA [38]	Linear	Maximize variance using orthogonal transformation	Preserves global structure, fast, interpretable	Linear relationships, outlier sensitive
ISOMAP [39]	Nonlinear	Geodesic distances via neighborhood graphs	Preserves global non-linear structure	Sensitive to noise, distorts local structure
t-SNE [40]	Nonlinear	KL divergence of pairwise similarity distributions	Preserves local structure	Can lose global structure, non-deterministic
UMAP [41]	Nonlinear	Manifold learning based topological method	Preserves local and some global structure	Parameter sensitive, global distortions
PCUMAP [37]	Nonlinear	UMAP with additional global structure objective based on Spearman’s and Pearson’s coefficients	Preserves both local and global relationships	Parameter sensitive

#### Evaluation metrics.

The goal of dimensionality reduction is to obtain a visually interpretable representation of high-dimensional data that preserves as much information as possible from the original data. We created a decision framework to comprehensively evaluate the performance of the dimensionality reduction methods using multiple metrics, focusing on topological preservation (local and global), and taking advantage of the expert-labels to gain further insights into the data structure across dimensionality reduction methods. This enabled us to identify the representation that best balanced structural fidelity and biological interpretability, providing a principled basis for selecting an embedding method rather than relying on heuristic or visual judgment alone.

#### A. Local Metrics.

Local structure preservation metrics evaluate how well the structure of a low-dimensional embedding matches that of the original high-dimensional embedding [42]. We use multiple common local structure preservation metrics, which are detailed below. For dendritic spine morphological modeling, local structure preservation is prioritized over global structure, since the aim is morphological subtype detection and fine-grained transition analysis. Fine-grained details are better preserved if local neighborhood distortions are low [40].

- **Trustworthiness score (T(k))** measures how well the local structure of the original data is maintained in low-dimensional space, where T(k)∈[0,1]. [43]. This metric penalizes any points that are nearest neighbors in the low-dimensional space but far away in the original high-dimensional space. Trustworthiness is defined as $T(k)=1-\frac{2}{nk(2n-3k-1)}\sum_{i=1}^{n}\sum_{j\in {𝒩}_{i}^{\text{embed}}}\max(0,r(i,j)-k)$, where *n* is the total number of samples (spines), *k* is the number of nearest neighbors considered, ${𝒩}_{i}^{\text{embed}}$ are the *k* nearest neighbors in the output space, and *r*(*i*,*j*) is the rank of each sample *j* in the original space. We considered *k* = 10 nearest neighbors in our implementation. A very small *k* can be prone to noise or underfitting, so setting *k* = 10 ensures we are still examining local relationships while mitigating errors due to noise. S6 Table in S1 File details the impact of varying *k* within the range 5 ≤ *k* ≤ 50 on the result.
- **Local Continuity Meta Criterion (LCMC)** measures the average overlap of the *K*-nearest neighborhoods of a point in the high-dimensional data and low-dimensional space [44], where LCMC∈[0,1]. It helps assess how well local relationships between points are preserved in low-dimensional space. *LCMC*(*k*) is defined as $\frac{1}{kn}\sum_{i=1}^{n}\left|{𝒩}_{i}^{\text{orig}}\cap {𝒩}_{i}^{\text{embed}}\right|$, where *n* is the number of samples (spines), *k* is the number of nearest neighbors, ${𝒩}_{i}^{\text{orig}}$ are the *k* nearest neighbors in the original feature space, and ${𝒩}_{i}^{\text{embed}}$ are the *k* nearest neighbors in the low-dimensional feature space. We use *k* = 10 to observe local-scale effects while mitigating noise. S6 Table in S1 File details the impact of varying *k* within the range 5≤k≤50 on the result.
- **Local Structure Preservation Score.** We compute an overall local structure preservation score that considers these metrics, using the average of the Trustworthiness and LCMC scores [37]:

$$\begin{array}{c} LS=0.5\times T(k)+0.5\times LCMC(k). \end{array}$$

We weight LS and GS equally to give equal preference to local and global structure preservation, but this weighting can be tuned to prioritize local or global structure for what is most appropriate for the researcher’s purposes.

#### B. Global Metrics.

Global structure preservation metrics measure how consistent point-pairwise distances are in the low-dimensional embedding compared to the original high-dimensional data- in other words, points that are close together or far apart in the original high-dimensional space should remain the same way in the low-dimensional embedding [42]. We implement two metrics, Pearson correlation and Spearman correlation, to evaluate the level of global distortion for each dimensionality reduction method.

- **Pearson’s correlation coefficient** measures how the pairwise distances between points varies in the original space and low-dimensional embedding space [42]. The score ranges from [−1, 1], where a negative score indicates negative linear correlation, 0 indicates no correlation, and a positive score indicates positive linear correlation. It is computed as the sample correlation coefficient: $r=\frac{\sum_{i=1}^{n}(x_{i}-\bar{x})(y_{i}-\bar{y})}{\sqrt{\sum_{i=1}^{n}(x_{i}-\bar{x}{)}^{2}}\sqrt{\sum_{i=1}^{n}(y_{i}-\bar{y}{)}^{2}}}$, where *x*_*i*_ and *y*_*i*_ represent the pairwise distances in the original and embedded space, and $\bar{x}$, $\bar{y}$ are their means.
- **Spearman’s rank correlation coefficient** measures the strength of the monotonic relationship between distances using the Pearson correlation on rank-transformed distances. The value ranges from [−1, 1], where −1 indicates a negative monotonic relationship, + 1 indicates a positive monotonic relationship, and 0 indicates no monotonic relationship. $\rho =\frac{\sum_{i=1}^{n}(r(x_{i})-\overset{―}{r(x)})(r(y_{i})-\overset{―}{r(y)})}{\sqrt{\sum_{i=1}^{n}(r(x_{i})-\overset{―}{r(x)}{)}^{2}}\sqrt{\sum_{i=1}^{n}(r(y_{i})-\overset{―}{r(y)}{)}^{2}}}$ where *r*(*x*_*i*_) and *r*(*y*_*i*_) are the ranks of the original and embedded pairwise distances, and $\overset{―}{r(x)}$, $\overset{―}{r(y)}$ are their means.
- **Global Structure Preservation Score.** We compute an overall global structure preservation score for ease of interpretability, using the average of the Pearson’s and Spearman’s coefficient values [37]:

$$\begin{array}{c} GS=0.5\times r+0.5\times \rho . \end{array}$$

#### C. Overall Structure Preservation Score.

We compute an overall structure preservation score for each dimensionality reduction method, using a weighted sum of the local and global structure preservation scores. The SPS is defined below, where *w*_LS_ is the weight of the local structure preservation score, *w*_GS_ is the weight of the global structure preservation score, and $w_{LS}+w_{GS}=1$.


$$\begin{array}{c} SPS=w_{LS}\cdot LS+w_{GS}\cdot GS, \end{array}$$

The results presented use $w_{LS}=0.5$ and $w_{GS}=0.5$ to give equal preference to local and global structure preservation. We also performed a sensitivity analysis to determine how the weighting of LS and GS impacts the selection of dimensionality reduction method (See S7 Table in S1 File). Depending on the researcher’s goals, they can tune this metric to prioritize local or global structure for visualization.

#### D. Biological Transition Score (BTS).

We additionally aim to establish a metric that can provide insight into the biological relevance of the data in low-dimensional space, focusing on the transition pattern of the data. It relies on annotated labels, but is used only to compare dimensionality reduction methods, not to drive clustering. This approach provides valuable insight into local relationships between spines in the low-dimensionality model, as well as a method of comparison of results across all dimensionality reduction techniques based on biological priors.

We developed BTS, which is a metric that provides a scalar value explaining how well each dimensionality reduction method preserves known biological relationships between different spine types. This metric is based on known literature about the plasticity of dendritic spines. Notably, a study from Bourne *et al*. examines the morphological transitions between “mushroom”, “stubby”, and “thin” spine types [45]. Their findings show that thin spines are highly plastic, and either mature into mushroom spines or are eliminated. Mushroom spine types are typically highly stable. Most immature dendritic spines are stubby. During long-term potentiation, stubby and thin spines can evolve into mushroom spines, while during long-term depression, stubby spines retract or shrink. However, stubby spines do not commonly evolve directly to thin spines [46]. We applied this domain knowledge toward creating a metric for biological relevance, where common spine transitions are rewarded, and uncommon spine transitions are penalized. Note that BTS is a comparative, heuristic metric for assessing preservation of biologically plausible transitions relatively across embeddings, rather than being a direct measurement of biological transition probabilities.

The score is calculated using both Euclidean Distances and Cosine Distances. Linear methods like PCA prioritize preserving global linear distance, which aligns with Euclidean distance metrics, potentially biasing evaluation in their favor. For this reason, including cosine distance, which measures angular relationships in the feature space, helps assess the preservation of local orientation and offers a more comprehensive view across both linear and nonlinear dimensionality reduction methods [38,47]. We defined BTS as


$$\begin{array}{c} BTS=\frac{\sum_{i=1}^{n}\sum_{j\in {\text{NN}}_{i}}w_{type_{i},type_{j}}\cdot \frac{1}{\log(d_{ij}+1)}}{\sum_{i=1}^{n}\sum_{j\in {\text{NN}}_{i}}w_{type_{i},type_{j}}}, \end{array}$$

where *w*_*i*,*j*_ is the weight of transitions between spine types *i* and *j* based on biological plausibility, *d*_*ij*_ is the distance between spines *i* and *j* in the low-dimensional space, and *NN*_*i*_ is the set of nearest neighbors of spine *i*.

Because there is no widely accepted quantitative model describing “transition rates” between spine morphologies, the transition weights used in BTS rely on biologically motivated approximations. The transition weights were assigned as follows, where within-spine clusters are assigned the highest score, common transitions are assigned a score between 0 and 1, and uncommon transitions are penalized with a score of 0. We use:

- Same-type transitions (Mushroom→Mushroom, Stubby→Stubby, Thin→Thin): 1.0;
- Mushroom↔Thin or Mushroom↔Stubby: 0.5;
- Thin↔Stubby: 0.0.

To assess the robustness of the BTS to assumptions about spine transition likelihoods, we evaluated a range of biologically plausible transition-weighting schemes (see S8 Table in S1 File). Across all weighting schemes, within-type transitions (e.g., Mushroom→Mushroom, Stubby→Stubby, Thin→Thin) were assigned the highest weight, reflecting local morphological similarity. The weight assigned to biologically common cross-type transitions, (e.g., transitions between Mushroom↔Thin spines and Mushroom↔Stubby spines) was varied from low to high values to test sensitivity to the strength of these relationships. Finally, transitions between Thin↔Stubby spines, which are rarely observed, were either fully penalized or assigned small nonzero weights to examine the impact of soft vs. hard penalties on uncommon transitions. Combining these weighting schemes span a broad but biologically informed space of transition assumptions, to evaluate whether comparisons remain stable under potential variations of prior biological knowledge.

### Probabilistic clustering to model spine shape transitions

#### Probabilistic clustering techniques.

After obtaining a representation of dendritic spine morphology that preserves both local and global structural relationships, we aimed to identify morphological groups based solely on their feature relationships while also capturing transitional phenotypes and quantifying uncertainty, aspects that are often overlooked in the traditional discrete spine classification scheme. To achieve this goal, we first determine the optimal number of spine type groupings for a given dataset, then test three clustering methods- Hierarchical Clustering, Fuzzy C-Means, and Gaussian Mixture Models- using quantitative metrics measuring cluster structure and spread. Each clustering method provides a different perspective and potential benefit when modeling spine structure and transitional shapes, summarized in Table 2. To avoid overfitting and ensure reproducibility, clustering algorithms were run using default parameter settings from their respective implementations, except where explicitly noted.

**Table 2:** Comparison of Clustering Methods for Dendritic Spine Transition Modeling.

Method	Clustering Type	Probabilistic	Transition Modeling	Structure Interpretability
Hierarchical [48]	Hard	No	Focus on structure preservation	Very high
FCM [49]	Soft	Degree-based	Very high	Medium
GMM [50]	Soft	Yes	High	Medium

A. **Hierarchical Clustering.** We implemented agglomerative hierarchical clustering using Ward’s linkage and Euclidean distance in the low-dimensional feature space [48]. This method results in each data point being assigned a hard membership label according to its cluster, without probabilistic insights. The dendrogram structure of hierarchical clustering can offer valuable insight into nested cluster relationships and structural similarity. While not probabilistic, hierarchical clustering was included to evaluate the extent to which strict structural organization aligns with continuous morphological transitions [10]. Starting with each point in its own cluster, hierarchical clustering continually merges the two closest clusters aiming to minimize the within-cluster variances based on the following equation: $D(A,B)=\frac{\left|A|\;|B\right|}{\left|A|+|B\right|}\;\|\mu_{A}-\mu_{B}{\|}^{2}$, where *A*, *B* are the clusters being merged, |*A*|, |*B*| are the number of elements in clusters *A* and *B*, $\mu_{A},\mu_{B}$ are the mean vectors of clusters *A* and *B*, and *D*(*A*, *B*) is the distance between clusters using Ward’s criterion.
B. **Fuzzy C-Means (FCM).** To allow for partial membership of spines across clusters, we applied FCM, a soft clustering technique that assigns a membership degree between 0–1 to each point for each cluster [49]. This method was chosen for its potential suitability in modeling transitional spine morphologies, since spines at intermediate stages may not belong exclusively to a single class, and probabilistic scoring of spine types could help identify transitional shapes [10]. We used a fuzzifier parameter *m* = 2, and clusters were formed based on minimizing the weighted within-cluster distances. FCM minimizes the following objective function: $J_{m}=\sum_{i=1}^{N}\sum_{k=1}^{K}u_{ik}^{m}\;\|{𝐳}_{i}-c_{k}{\|}^{2}$. Membership degrees *u*_*ik*_ are updated iteratively using the equation: $u_{ik}=\frac{1}{\sum_{j=1}^{K}{\left(\frac{\left\|{𝐳}_{i}-c_{k}\right\|}{\left\|{𝐳}_{i}-c_{j}\right\|}\right)}^{\frac{2}{m-1}}}$. ${𝐳}_{i}\in {\mathbb{R}}^{d}$ is the embedding of spine *i*, $c_{k}\in {\mathbb{R}}^{d}$ is the centroid of cluster *k*, $u_{ik}\in [0,1]$ is the degree of membership of spine *i* in cluster *k*, *N* is the total number of spines, *K* is the number of clusters, and *m* is the “fuzzifier” controlling softness.
C. **Gaussian Mixture Models (GMM).** Finally, we utilized GMM to capture both soft cluster assignments and data density distributions. GMM assumes that data points are generated from a mixture of multivariate Gaussian distributions, and provides posterior probabilities for each spine’s membership in every cluster [50]. Similar to FCM, this approach provides probabilistic scoring for each data point’s membership, but has the additional benefit of modeling uncertainty and transition likelihood. We used the Expectation-Maximization (EM) algorithm for parameter estimation. GMM models the data distribution as a weighted sum of multivariate Gaussians using the following equation: $p({𝐳}_{i})=\sum_{k=1}^{K}\pi_{k}\;𝒩({𝐳}_{i}|\mu_{k},\Sigma_{k})$. The soft assignment of point **z**_*i*_ to component *k* is $\gamma_{ik}=\frac{\pi_{k}\;𝒩({𝐳}_{i}|\mu_{k},\Sigma_{k})}{\sum_{j=1}^{K}\pi_{j}\;𝒩({𝐳}_{i}|\mu_{j},\Sigma_{j})}$. $\pi_{k}$ is the mixture weight of component *k*, with $\sum_{k=1}^{K}\pi_{k}=1$, $\mu_{k}$ is the mean vector of Gaussian component *k*, $\Sigma_{k}$ is the covariance matrix of component *k*, 𝒩(·∣μ,Σ) is the multivariate normal density function, and $\gamma_{ik}$ is the posterior probability that point *i* belongs to cluster *k*.

#### Cluster number selection.

Selecting an appropriate number of clusters is a critical step for modeling dendritic spine morphology, especially given the continuous and transitional nature of spine shapes. To determine the optimal number of clusters for each clustering method, we employed two complementary methods that assess cluster separability and geometric structure.

A. **Silhouette Analysis.** Silhouette analysis was used to evaluate clustering performance across a range of candidate cluster numbers *k*. For each value of *k*, we computed the mean Silhouette score across all samples, which reflects the balance between intra-cluster cohesion and inter-cluster separation. These scores were plotted as a function of *k*, and the optimal number of clusters was selected based on the first peak, which results in a solution with strong separability while avoiding unnecessary complication or fragmentation of the data [51].
B. **Convex Hull (CHull) Method.** To further inform cluster number selection, we applied the CHull method, which evaluates the trade-off between cluster compactness and model complexity. The CHull method constructs a convex hull to capture within-cluster dispersion and between-cluster separation, where the “elbow” on the plot represents a solution that balances cluster separation with model complexity [52]. This method is particularly suited for identifying structural organization in datasets with overlapping or continuous cluster structure, which is useful for our dendritic spine application where morphologies, and therefore clusters, are continuous.

#### Evaluation metrics.

To evaluate the clustering results of these three methods and determine which produces the best representation of dendritic spine shape, we use a combination of hard and soft clustering metrics. The hard clustering metrics focus on separability of the clusters in the low-dimensional space, which gives insights into the general spread of the data, allowing us to determine if there are significant differences between methods. We additionally evaluate multiple soft clustering methods, which measure membership confidence. This gives us insights into the confidence of cluster assignments across methods.

#### A. Hard metrics.

Hard membership metrics for evaluating clustering models assume that objects either belong or do not belong to a certain cluster, based on inter-cluster and within-cluster similarities. We use multiple common hard clustering metrics, which are detailed below [52].

- **Silhouette Score** is a measure of the mean intra-cluster distance and nearest-cluster distance for each sample [51]. This metric was used both to determine the optimal number of clusters in the low-dimensional feature space using each clustering method, and to evaluate cluster separability. The goal of this metric is to evaluate how similar a spine is to its own cluster compared to other clusters. The score ranges between [−1, 1], where a negative score indicates the spine is likely assigned to the wrong cluster, while a score of “1” indicates perfect cluster assignment. The Silhouette score is computed using $s(i)=\frac{b(i)-a(i)}{\max\{a(i),b(i)\}}$, where *a*(*i*) is the average distance from a sample to all other samples in the cluster, and *b*(*i*) is the average distance from sample *i* to all samples in the nearest other cluster.
- **Davies-Bouldin (D-B) Score** measures the average similarity of each cluster with its most similar cluster, where similarity is defined as the ratio of within-cluster distances to between-cluster distances [53]. Clusters that are farther apart will have a lower (better) score, with the minimum possible score of 0. D-B is defined as $D-B=\frac{1}{n}\sum_{i=1}^{n}\max_{j\neq i}\left(\frac{s_{i}+s_{j}}{d_{ij}}\right)$, where *n* is the number of clusters, *s*_*i*_ is the average distance of all points in a cluster to its centroid, and *d*_*ij*_ is the distance between the centroids of clusters *i* and *j*.
- **Calinski–Harabasz (C-H) Index** measures the ratio of between-cluster dispersion to within-cluster dispersion, providing an indication of how well clusters are separated and how compact they are [54]. A higher score indicates better-defined, more separated clusters. The C-H Index is defined as $C-H=\frac{\text{Tr}(B_{k})}{\text{Tr}(W_{k})}\times \frac{n-k}{k-1},$ where *n* is the number of samples and *k* is the number of clusters, Tr(*B*_*k*_) is the trace of the between-cluster dispersion matrix, and Tr(*W*_*k*_) is the trace of the within-cluster dispersion matrix.

#### B. Soft metrics.

For probabilistic clustering and transitional modeling, soft clustering metrics are useful for understanding the strength or confidence to which a sample belongs to a group [49]. Since dendritic spines exist on a continuum, explicitly modeling probabilistic memberships to each group can be used to inform whether a spine is in a transitional state. We use multiple common soft clustering metrics to evaluate our models.

- **Entropy** measures the average uncertainty in the soft cluster assignments by quantifying how uniformly each sample’s membership is distributed across clusters. For a sample *i* with membership probabilities $p_{i1},\ldots ,p_{iK}$, the Shannon entropy is defined as $H_{i}=-\sum_{k=1}^{K}p_{ik}\log p_{ik},$ and the **Average Entropy** over all *n* samples is $\frac{1}{n}\sum_{i=1}^{n}H_{i}=\frac{1}{n}\sum_{i=1}^{n}\left(-\sum_{k=1}^{K}p_{ik}\log p_{ik}\right).$ Lower values indicate higher certainty in assignments, and higher values reflect greater fuzziness or uncertainty in cluster membership [55].
- **Sharpness** measures the concentration of membership probabilities. Given the maximum possible entropy logK, the **Average Sharpness** is defined as $\frac{1}{n}\sum_{i=1}^{n}\left(1-\frac{H_{i}}{\log K}\right)=1-\frac{1}{n\log K}\sum_{i=1}^{n}H_{i}.$ The value ranges from [0, 1], where a value of 1 reflects maximum certainty while values closer to 0 reflect higher fuzziness [49].
- **Average Maximum Probability** measures how strongly each sample favors its most likely cluster, and is measured as the average of the maximum membership probabilities across all samples: $\frac{1}{n}\sum_{i=1}^{n}\max_{1\leq k\leq K}p_{ik}.$ Higher values indicate that data tends to have a single dominant cluster membership, implying greater membership confidence. Lower values suggest more fuzzy cluster assignments [49].

### Dendritic spine visualization and probabilistic clustering decision framework

**Fig 1:**
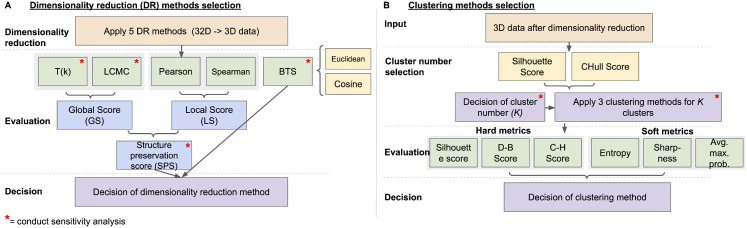
Schematic of decision approach for dimensionality reduction and clustering. **(A)** Five Dimensionality reduction methods are applied to the high-dimensional feature set, and their embeddings are evaluated using global structure metrics, local structure metrics, and BTS computed using Euclidean or cosine distances. These metrics are combined into the SPS metric, enabling users to select an embedding method suited to the structural properties of their dataset. **(B)** The chosen low-dimensional embedding is used to identify an appropriate number of clusters based on Silhouette and CHull scores. Three clustering algorithms are then applied using the selected cluster number. Hard metrics and soft metrics are used to evaluate clustering. Sensitivity analyses are performed for key parameters, indicated by a “*”.

Decision frameworks are valuable tools for visually mapping and simplifying complex problems to help guide users to a structured decision. To help guide the visual characterization of dendritic spine morphology, we developed the workflow presented in Fig 1. It is important to note that this approach is both modular and dataset-dependent: while metric definitions are fixed, there are hyperparameters, weights, and features that can be tuned using sensitivity analysis to optimize for the specific application. The workflow proceeds in three stages. First, we collect and verify quantitative morphological features that capture the geometric and intensity-based properties of dendritic spines. Next, we apply the dimensionality-reduction decision framework to evaluate multiple embedding methods using a set of local, global, and biologically informed metrics (Fig 1A). Finally, we use the clustering selection decision framework to assess candidate cluster numbers and clustering strategies, enabling probabilistic interpretation of transitional morphologies (Fig 1B).

In this study, we apply the approach to two labeled 2PLSM-imaged neonatal mouse hippocampal dendritic spine datasets to represent the spine morphological landscape and continuous and transitional spine morphologies in our data. We show how the approach can be used to choose an appropriate embedding and clustering method for a given dataset based on image resolution and resolvable features, structure preservation, interpretability, and biological relevance. To facilitate implementation, all code has been made available on GitHub and can be adapted for other dendritic spine studies.

## Results

### Evaluating dimensionality reduction methods using the decision framework identifies PCUMAP as the most suitable embedding for the dataset

**Fig 2:**
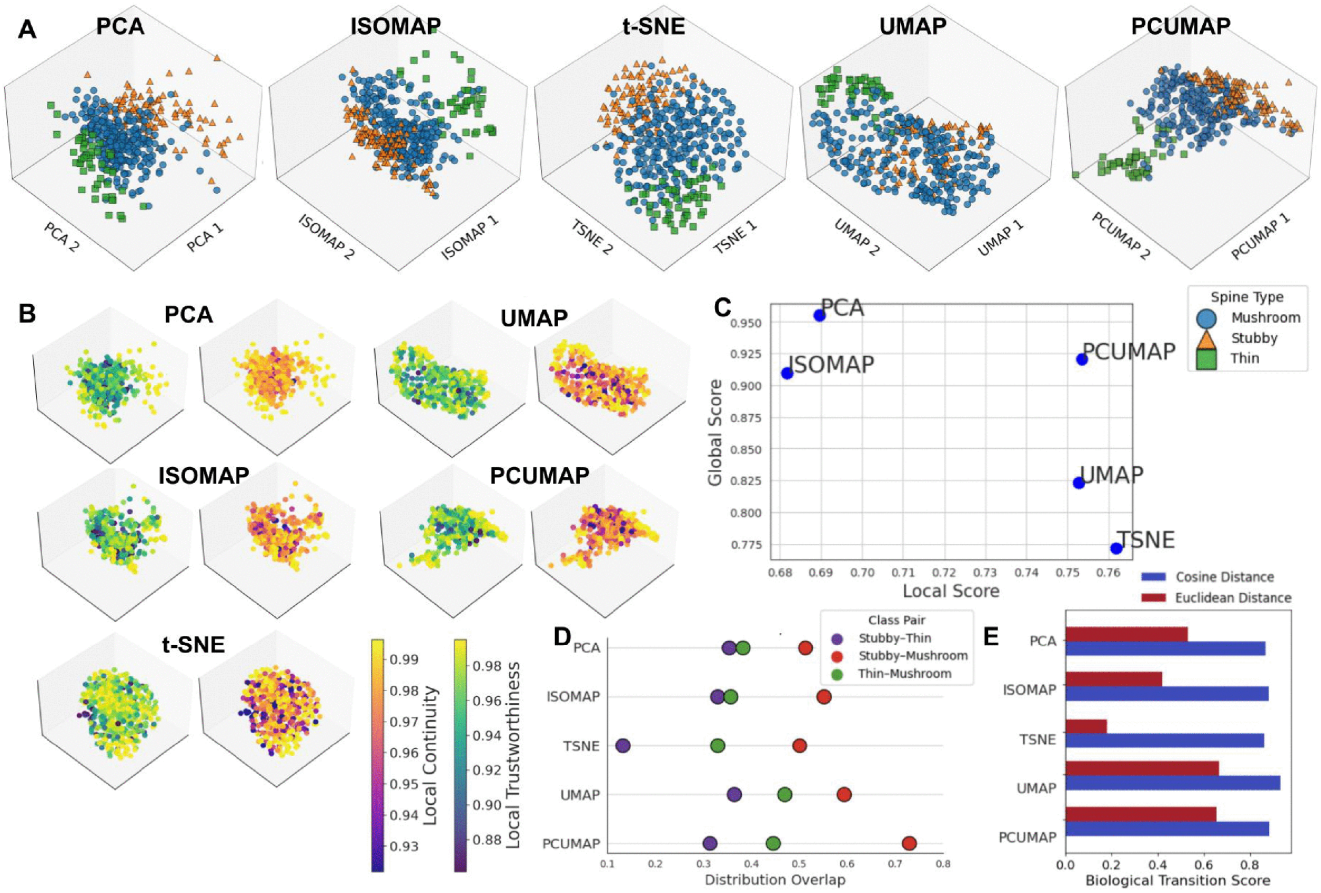
Result of dimensionality reduction of dendritic spine feature dataset using 5 dimensionality reduction methods. **A)** Data with expert labels (“mushroom”, “stubby”, “thin”) in low-dimensional space using 5 dimensionality reduction techniques. **B)** Comparison of local distortion reliability across five methods, using local continuity (how well neighbors in the low-dimensional space match neighbors in the original space), and trustworthiness (how well neighbors in the original space are maintained in the low-dimensional space). **C)** Local score (LS) vs. Global score (GS) across 5 methods; PCUMAP offers balance between GS and LS, followed by UMAP and PCA. **D)** The Kernel Density Estimation (KDE) distribution overlap, which estimates the probability density distribution of each group between clusters, is similar across all methods but lower for t-SNE, and we consistently saw a low distribution overlap between stubby and thin spines, and a high overlap between stubby and mushroom spines. **E)** BTS is highest for UMAP when using Euclidean distance, but is more consistent across methods using Cosine distance‌‌.

The results of applying the five dimensionality reduction techniques to the dendritic spine shape, intensity, and curvature feature dataset collected using 2PLSM dendritic spine images from Ghani et al. [31] are shown in Fig 2. The high-dimensional feature data were reduced into three-dimensional projections for ease of visualization. The choice of embedding dimensionality is defended in S3 Table in S1 File, where we evaluated 2–5 dimensions across all dimensionality reduction methods and found that increasing dimensionality improved geometric preservation, while clustering quality peaked at 3 dimensions and remained stable. Next, we used our dimensionality-reduction decision framework to evaluate each method’s performance through both visual assessment and quantitative metrics, revealing distinct patterns in how each method preserves intrinsic feature structure and biological relationships.

Fig 2A shows the three-dimensional projections obtained from each dimensionality reduction method. The data points are colored according to manual ground-truth labels: green squares for thin spines, blue circles for mushroom spines, and orange triangles for stubby spines.

#### Local and global structure preservation.

Fig 2B presents local continuity and local trustworthiness. t-SNE, UMAP, and PCUMAP show high continuity and trustworthiness, indicating strong preservation of local structure. PCA maintains original neighborhoods but introduces spurious neighbors in the low-dimensional space, resulting in lower trustworthiness. ISOMAP performed the weakest across local metrics. Table 3 reports the trustworthiness and continuity scores for each method, used to compute LS. S6 Table in S1 File details a sensitivity analysis that varies the number of nearest neighbors used in the calculation of local metrics, showing that the relative ranking of dimensionality reduction methods remains stable across a wide range of neighborhood sizes.

**Table 3:** Comparison of five dimensionality reduction methods across local and global structure preservation metrics. Local metrics include *T*(*k*) and LCMC, which were combined into an overall LS score. Global metrics include Pearson’s and Spearman’s coefficients, which were combined into an overall GS score. SPS was computed using a weighted sum of LS and GS. BTS was computed using both Euclidean and Cosine distances to represent each method’s preservation of biologically relevant relationships based on expert annotations. The final column reports the average overlap (AO) across all expert-label class pairs (lower indicates better class separation). Bold values indicate the best-performing method for each metric, while underlined values denote the second-best result.

Method	Local			Global			SPS	BTS		AO
	T(k)	LCMC	LS	Pearson	Spearman	GS		Euc.	Cos.	
PCA	0.935	0.444	0.690	**0.961**	**0.949**	**0.955**	0.822	0.536	0.867	0.412
ISOMAP	0.928	0.435	0.682	0.920	0.900	0.910	0.796	0.421	0.881	0.470
t-SNE	**0.975**	**0.549**	**0.762**	0.770	0.774	0.772	0.767	0.180	0.882	0.343
UMAP	0.962	0.543	0.753	0.823	0.823	0.823	0.788	**0.670**	0.932	0.479
PCUMAP	0.966	0.545	0.755	0.926	0.914	0.920	**0.838**	0.656	**0.903**	0.501

Table 3 also presents global structure metrics, Pearson and Spearman correlations, which are used to compute the GS. These results are visualized in Fig 2C, which illustrates how each method balances preservation of local and global structure. PCA shows strong global preservation but comparatively high local distortion. UMAP and t-SNE preserve local structure well but have reduced global correlation. PCUMAP provides the most balanced performance, retaining both local continuity and broader global trends.

#### Structure Preservation Score (SPS).

We computed the overall SPS for each dimensionality reduction method as defined in the Methods section (Table 3). When equal weighting was applied $(w_{LS}=0.5$ and $w_{GS}=0.5)$, PCUMAP achieved the highest SPS among the evaluated methods. To evaluate whether this result depended on the chosen weighting, we performed a sensitivity analysis by varying the weighting parameter between 0.3 and 0.7 to prioritize local or global structure (S7 Table in S1 File). Across all weighting schemes tested, including cases emphasizing primarily local or primarily global structure, PCUMAP consistently achieved the highest SPS. PCA and UMAP generally followed in performance, while ISOMAP produced the lowest SPS values under these criteria. These results suggest that the superior performance of PCUMAP on this dataset is not dependent on a specific weighting choice, but reflects robust preservation of both local and global structure.

#### Distribution and Biological Transition Modeling.

Fig 2D shows distribution overlaps for the three manually labeled classes (Stubby–Thin, Stubby–Mushroom, and Thin–Mushroom). All methods show low overlap between stubby and thin spines. t-SNE yields the lowest overlaps overall (reported in Table 3 as AO), while PCUMAP shows higher overlap across groups.

BTS was evaluated using both Euclidean and cosine distances to capture complementary aspects of feature relationships (See Methods and S8 Table in S1 File for more details on this metric). Using Euclidean distance, UMAP and PCUMAP achieve the highest BTS values, followed by PCA, while ISOMAP and t-SNE show lower alignment with expected biological relationships. When using the cosine distance, all the methods show a high BTS, suggesting that the angular relationships between feature vectors reflect biologically significant transitions more consistently than the absolute Euclidean distances (Table 3). The BTS calculation incorporates biologically motivated transition weights derived from prior literature on dendritic spine remodeling [45,46]. Because these weights are necessarily approximate, we assessed the robustness of BTS by performing a sensitivity analysis across alternative biologically plausible weighting schemes (S8 Table in S1 File), which showed that relative method rankings remain stable across a wide range of parameter choices.

#### Determination of most suitable dimensionality reduction method.

Among the dimensionality reduction approaches evaluated, PCUMAP achieves the most balanced preservation of the local structure, the global structure, and the BTS in this data set. Therefore, we selected PCUMAP for subsequent clustering and transition modeling steps.

#### Clustering in the selected embedding space reveals a continuum of dendritic spine morphologies.

We apply three clustering techniques (Hierarchical Clustering, FCM, and GMM) to the PCUMAP data projection, obtaining a representation of spine connections that uses nonlinear combinations of features to determine groupings (Fig 4A).

**Fig 3:**
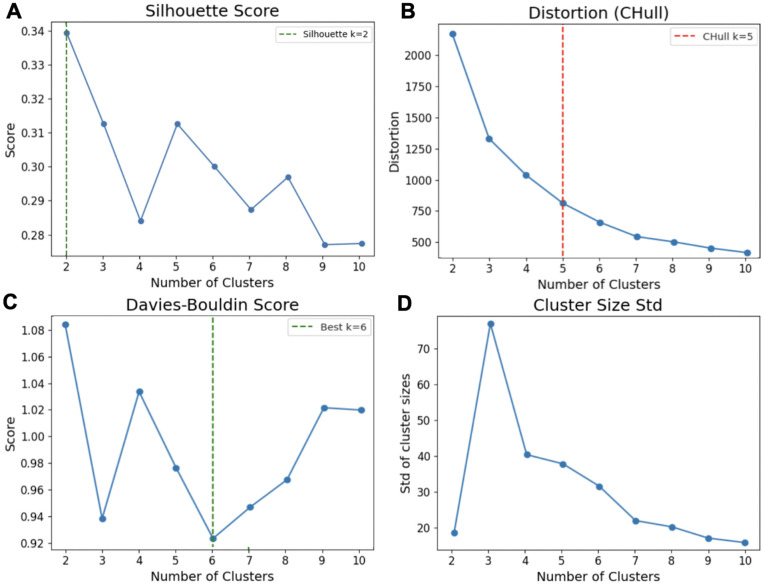
Cluster-number selection diagnostics for FCM clustering on the 3D PCUMAP embedding. Cluster solutions for *K* = 2–10 were evaluated using complementary structural criteria, including A) the Silhouette score (cluster compactness and separation), B) distortion-based CHull analysis (convex hull elbow), C) the D-B index (cluster overlap; lower values indicate better separation), and D) the standard deviation of cluster sizes (partition balance). The Silhouette score peaked at *K* = 2, reflecting coarse partitioning into broad groups. Separation-based metrics (D-B index) favored slightly larger values (K≈6), while CHull distortion analysis identified an elbow at *K* = 5, indicating diminishing returns in compactness improvement beyond this point. Cluster size diagnostics revealed substantial imbalance at *K* = 3, consistent with under-segmentation, whereas larger *K* values progressively subdivided the embedding structure. Taken together, these complementary metrics support *K* = 5 as a balanced compromise between under-resolution at small *K* and over-fragmentation at larger ***K*.**

#### Decision of cluster number.

To determine the appropriate number of clusters for this dataset and examine how dendritic spine morphologies distribute across groups, we evaluated cluster solutions for K = 2–10 using multiple complementary criteria. Specifically, we assessed the Silhouette score [51], the CHull method [52], the D-B index, and cluster size balance (standard deviation of cluster sizes) (Fig 3). These metrics did not converge to a single optimal K, which is expected given the continuous, branching structure of the embedding. The Silhouette score peaked at K = 3, reflecting coarse partitioning into broad groups (Fig 3A). However, cluster size diagnostics revealed substantial imbalance at K = 3, indicating under-resolution of morphological heterogeneity. In contrast, separation-based metrics (e.g., D-B) continued to improve until K = 6, suggesting additional substructure (Fig 3C). The CHull distortion analysis identified an elbow at K = 5, corresponding to a point of diminishing returns in compactness improvement (Fig 3B). At K = 2, the embedding was divided into two broad lobes, merging morphologically distinct spine types and failing to resolve transitional structure. At K = 8, clusters fragmented in the embedding, producing smaller and less interpretable subgroups without substantial gains in structural separation (S10, S11 Tables in S1 File). Taken together, these analyses indicate that K = 5 provides the most balanced solution, resolving meaningful substructure beyond the canonical three-class organization while avoiding over-fragmentation at higher K.

#### Hard metrics.

We evaluated hierarchical clustering, FCM, and GMM using hard clustering metrics that quantify cohesiveness and separability (Table 4). All three methods produced reasonably cohesive and well-separated clusters, with FCM showing slightly stronger overall performance (Silhouette score = 0.351). Both FCM and hierarchical clustering yielded lower D-B scores (0.957 and 0.964, respectively), indicating reduced similarity between clusters compared to GMM. FCM also achieved the highest C-H value (381.61), reflecting compact clusters with strong separation.

**Table 4:** Comparison of clustering methods applied to PCUMAP dendritic spine shape embedding, using both hard and soft clustering metrics. Hard clustering metrics (Silhouette score, C-H Index, and D-B Index (D-B)) evaluate the quality of discrete cluster assignments. Soft clustering metrics (average entropy, sharpness, and maximum membership) assess uncertainty and confidence in probabilistic cluster memberships. Bold values indicate the best-performing method for each metric.

Method	Hard Metrics			Soft Metrics – FCM and GMM only		
	Silhouette	C-H Score	D-B Score	Avg Entropy	Avg Sharpness	Avg Max Prob
Hierarchical	0.324	336.84	0.964	–	–	–
FCM	**0.351**	**381.61**	**0.957**	5.397	0.112	0.970
GMM	0.281	280.85	1.047	0.239	0.852	0.905

#### Soft metrics.

When comparing FCM’s performance to GMM on the soft clustering metrics, FCM had a “fuzzier” result compared to GMM, with a higher average entropy and lower average sharpness. This indicates that while the dominant cluster assignment for the spines was well separated, individual spines exhibited more distributed membership among the clusters under FCM than under GMM. Probabilistic spread is consistent with the biological continuum of dendritic spine morphologies, allowing individual spines to belong to multiple clusters with varying degrees of membership, better capturing the reality that the boundaries between classical categories are gradual rather than discrete.

#### Spine distributions across clusters.

**Fig 4:**
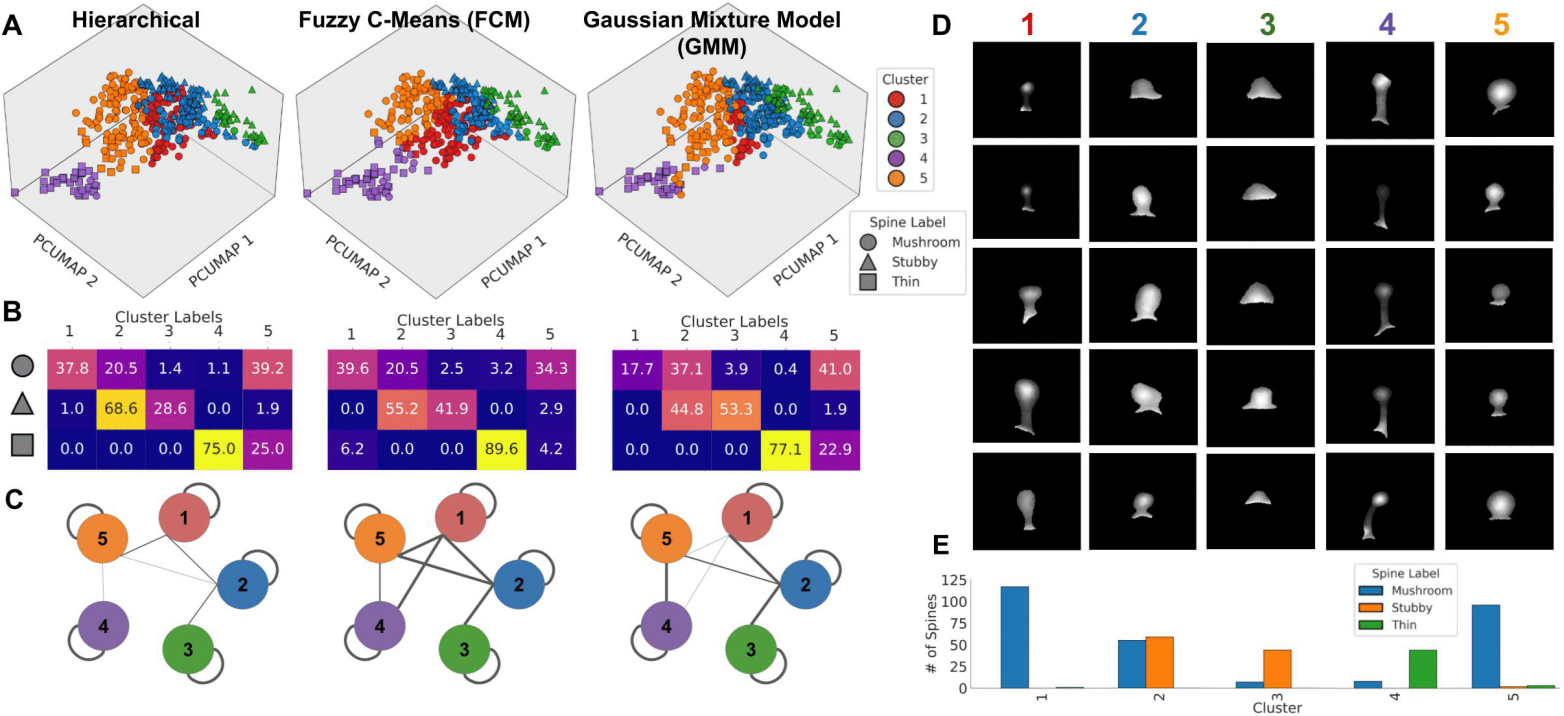
Result of clustering of dendritic spine feature data in PCUMAP space using 3 clustering methods. **A)** Result of clustering using 3 methods on PCUMAP projection of dendritic spine data in 3 dimensions. **B)** Distribution of mushroom, stubby, and thin spines across the 5 clusters for each clustering method. Mushroom spines are more distributed across the five classes, while thin and stubby spines are generally well-represented within just one to two clusters. **C)** Transitional graphs showing the distribution of each cluster’s nearest neighbors. The probabilistic clustering methods (FCM, GMM) show stronger edges between clusters. **D)** Example spines in each cluster from FCM clustering. **E)** Distribution of spine types across clusters for FCM clustering: mushroom spines are primarily distributed across clusters 1, 2, and 5. Stubby spines are primarily distributed across clusters 2 and 3. Thin spines are primarily contained in cluster 4.

As shown in the heat maps in Fig 4B, which compare cluster assignments with expert-provided labels (“mushroom”, “stubby”, “thin”), all three spine types are distributed across the five clusters to different extents. Thin and stubby spines are concentrated primarily within one to two clusters, whereas mushroom spines are distributed across multiple clusters in varying proportions depending on the clustering algorithm. This distribution suggests that mushroom spines themselves represent a broad morphological spectrum rather than a homogeneous category, and traditional classification methods fail to capture the variations within this spine type. Given that mushroom spines are associated with structural stability and long-term memory, capturing the diversity of their morphologies is essential for linking structure to synaptic function.

The connectivity graphs (Fig 4C) illustrate the Euclidean nearest neighbors of each spine, categorized by cluster, in the 3D PCUMAP embedding, providing further insight into local neighborhood relationships consistent with potential intermediate organization. FCM clustering captures more overlap between neighboring clusters. This pattern aligns with the biological expectation that dendritic spines remodel through gradual morphological changes rather than discrete jumps. In comparison, hierarchical clustering produces more rigid connectivity patterns with fewer intermediate states.

#### Merit of 5 clusters compared to 3 clusters or expert labels.

To determine whether increasing the number of clusters actually captures meaningful morphological heterogeneity, we performed an additional sensitivity analysis directly comparing data structure preservation of (1) expert labels, (2) clustering at K = 3, corresponding to the canonical “mushroom,” “stubby,” and “thin” classes, and (3) K = 5, using identical evaluation metrics, where K is the number of clusters (see S9 Table in S1 File). This comparison is necessary to assess whether increasing the number of clusters leads to artificial fragmentation of existing spine categories or instead reveals stable, interpretable substructure within them. As expected, because K = 3 aligns with the three-class structure of the expert annotations, it showed slightly higher agreement with the ground-truth labels. However, clustering at K = 5 maintained comparable cluster compactness and separation, as indicated by similarly high Silhouette, C-H, and D-B scores. Additionally, K = 5 clustering achieved equal or stronger structural separation compared to the expert ground-truth labels evaluated directly in the same embedding across all metrics, suggesting that the clustering-based representations capture morphological structure that is not fully reflected by the discrete expert classification. These results indicate that increasing the number of spine-type categories can preserve the structural organization of canonical spine types while also resolving additional substructure within traditional categories, providing a more refined and biologically meaningful representation of dendritic spine morphology rather than simply being an artifact of over-segmentation.

#### Dataset imbalance analysis.

It is also important to note that the dataset is imbalanced in spine types, with mushroom spines comprising the majority of the dataset, followed by stubby spines, and fewer thin spines. While this does align with naturally occurring distributions of spine types reported in prior studies [9,45], this imbalance could potentially influence clustering behavior. To assess the robustness of our results to spine-type imbalance, we performed additional sensitivity analyses comparing clustering outcomes on the original dataset to the result on a balanced subset with equal representation of each spine type (S14 Table, S11 Fig in S1 File). We found that across all clustering methods, balancing the dataset led to more uniform cluster sizes and similar clustering confidence compared to the original dataset, as evidenced by reduced cluster-size variability and stable membership certainty. However, the qualitative relationships between clusters and spine types were preserved after balancing: thin spines remained well separated, primarily in their own cluster, and there was still little to no overlap between stubby and thin spines within the same cluster. These analyses indicate that the observed cluster organization presented here reflects intrinsic morphological structure captured by our method, and is not only driven by spine-type imbalance.

#### Determination of clustering method and cluster interpretation.

Applying FCM clustering to PCUMAP spine characteristics revealed intermediate morphologies between stubby, mushroom, and thin. Fig 4E shows the distribution of spine labels within each cluster. Fig 4D presents representative spines from each group. While we saw a distribution of spine types across each of the clusters, we observed several consistent feature-based trends (see S10 Fig in S1 File):

- **Cluster 1**: primarily mushroom spines with larger heads and longer necks.
- **Cluster 2**: stubby and mushroom spines with short, wide necks, high width-to-length ratios, and homogeneous intensity.
- **Cluster 3**: predominantly stubby spines with little to no neck.
- **Cluster 4**: primarily thin spines with long necks and elevated intensity and textural features (fractal dimension, skewness), indicating higher morphological irregularity.
- **Cluster 5**: primarily mushroom spines with short necks and circular, uniform intensity profiles.

A complete list of all spines and their cluster assignments is provided in S13 Table in S1 File. Together, these results demonstrate that FCM provides a flexible framework for quantifying graded cluster membership and intermediate morphologies within this dataset. Notably, similar mixed-membership structure was observed across all embeddings even with lower BTS (S12 Table in S1 File), indicating that this continuum-like organization is not unique to the selected embedding.

#### Insights from probabilistic cluster assignments.

**Fig 5:**
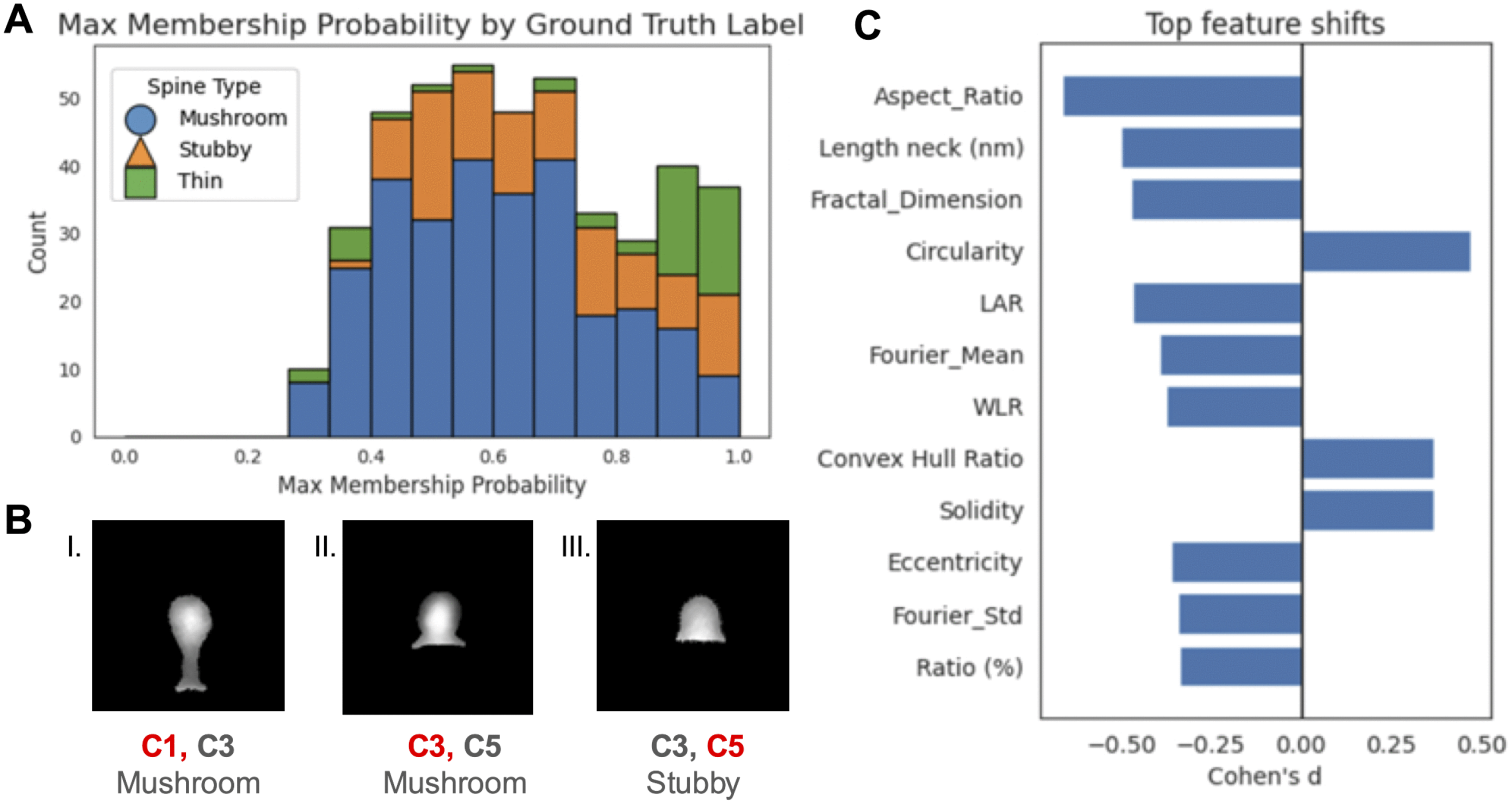
Insights from probabilistic cluster assignments. **(A)** Maximum membership probability for each spine, color-coded by annotated label. **(B)** Examples of spines with high membership in more than one cluster. The primary cluster assignment is annotated in red. **(C)** Top feature shifts using Cohen’s d metric. This shows which features were most associated with ambiguous spine types (max probability ≤ 0.6)‌‌.

Beyond the framework evaluation, applying the selected clustering method to this dataset revealed several biologically meaningful patterns. The distribution of maximum membership probabilities (Fig 5A) shows that mushroom and stubby spines often have uncertain assignments, whereas thin spines are more confidently classified. This pattern is consistent with existing literature, which finds thin spines to be more morphologically distinct, while mushroom and stubby spines represent a continuum of head and neck geometries [7]. The mild imbalance in the dataset, with more mushroom and stubby examples than thin, may also contribute to this spread.

Individual examples also highlight transitional morphologies. Fig 5B shows spines with high membership in multiple clusters. Spines I and II were labeled mushroom by the annotator, while spine III was labeled stubby. However, the clustering approach reveals that both spine II and III show high membership in clusters 3 and 5, revealing a morphological continuum of shape across these three spine types that does not reflect using the traditional hard classification scheme.

To characterize the morphology of these intermediate spines, we compared ambiguous and confident spines using Cohen’s d score (standardized difference between the mean feature value for confident vs ambiguous spines). For each spine, we quantified maximum cluster membership and normalized entropy, and defined ambiguous assignments as spines with maximum membership probability less than 0.60. We found that ambiguous spines tended to have lower elongation/neck-related features (e.g., aspect ratio, LAR, neck length, eccentricity) and higher compactness-related measures (e.g., circularity, solidity), suggesting that the continuum is expressed through gradual variation in shape compactness and elongation rather than abrupt transitions between fully separated classes (Fig 5).

These results support the view that dendritic spine morphology is best described as a continuous landscape of developmental and functional states rather than as a set of discrete categories defined by a limited number of shape-based criteria [9]. Across the evaluated embeddings and clustering strategies, we observe gradual transitions and substantial overlap between traditional spine classes, with probabilistic clustering revealing hybrid morphologies that are obscured by hard classification. By allowing individual spines to exhibit partial membership across multiple clusters, our framework captures intermediate and transitional forms that reflect smooth morphological progression rather than discrete boundaries. These findings demonstrate that the proposed modeling approach preserves the global organization of the traditional classifications while providing increased resolution of intra-class heterogeneity, offering a more nuanced and biologically faithful representation of dendritic spine morphological variation.

#### Generalization of decision framework to new datasets.

To assess the robustness of our decision framework across imaging conditions, we applied the same dimensionality reduction and clustering selection procedure to an independent lower-resolution dataset from Smirnov et al. [32] (S15-S16 Table, S12 Fig in S1 File). Despite differences in resolution and acquisition parameters, the systematic evaluation of structure-preservation metrics and clustering validity indices again identified a five-cluster solution as optimal. In this dataset, PCA was selected as the preferred dimensionality reduction method, reflecting the lower spatial resolution and reduced nonlinear structure. The emergence of five morphometric groupings across both datasets supports the generalizability of the proposed framework and suggests that the identified clustering structure is not specific to a single imaging modality or resolution.

## Discussion

### Significance and biological implications

By combining probabilistic clustering with a detailed set of quantitative morphological features, our analysis identifies biologically relevant spine groupings that align with established findings while also revealing intermediate forms not captured by traditional categorical schemes, improving our understanding of dendritic spine diversity. Our framework models how multiple features co-vary, creating a more holistic representation of dendritic spine diversity that would be difficult to distinguish without computational tools.

The findings from this study arise from applying our dimensionality reduction and clustering decision framework to this dataset of dendritic spines from early postnatal mouse CA1 hippocampal neurons. The continuum of spine shapes observed here is especially important given the highly dynamic nature of dendritic spines during early development. Recent studies show extensive dendritic spine remodeling occurs during the early postnatal stage in humans, with dendritic spine density peaking in humans around 3 weeks after birth [56], and continuing throughout adolescence before stabilizing during adulthood [57]. The mouse postnatal hippocampal CA1 region (from which the dendritic spine data was collected) undergoes similar rapid synaptogenesis and structural changes, and spines at this stage are highly plastic. In such a phase of high structural remodeling, many growing, transitional, and unstable spine forms exist. Rather than forcing these developing spines into rigid categories, our clustering model captures the incremental continuity of spine shape to observe the probabilistic shifts in spine features across clusters, creating a framework for tracking structural plasticity quantitatively.

The dataset contains more mushroom and stubby spines than thin spines, consistent with naturally occurring developmental distributions reported in prior studies [9,45]. Although the clustering algorithms used in this work are unsupervised (do not consider class labels), dataset imbalance could still, in principle, influence clustering results, leading to under-represented spine types being grouped together simply as a result of the data representation. To address this, we performed supporting analyses evaluating the impact of spine-type imbalance on clustering outcomes and cluster–label relationships (S14 Table and S11 Fig in S1 File). Across these analyses, the qualitative continuum patterns and transitional morphologies remained similar, reinforcing the interpretation that the structures identified by our framework reflect underlying biological variability rather than class-proportion artifacts.

Finally, this approach has implications for understanding the mechanisms of neurological disease by representing spines that occupy intermediate states, working toward a more fine-tuned understanding of how subtle changes in geometry can alter neural transmission. For example, certain conditions display an over- or under-representation of particular spine categories [6]. While static spine counts can be helpful for revealing broad shifts, a transitional spine type model allows researchers to track how dendritic spines move between states to potentially identify early-stage structural deviations associated with spine elimination or growth [4,6].

Overall, the decision framework presented here is a flexible approach for representing dendritic spine morphology. By emphasizing structure-preserving embeddings and probabilistic clustering, our approach enables researchers to capture subtle and biologically meaningful morphological continuity. In the future, this framework can be applied to other datasets or experimental conditions, with the potential to aid with quantification of structural plasticity, developmental trajectories, and early morphological signatures of neural disease. Broadly, the same decision approach can also be extended beyond dendritic spines to other biological structures that exhibit continuous morphological variation for data-driven characterization of shape dynamics.

### Conclusions and future work

Our decision framework and results on two dendritic spine datasets emphasize the value of using unbiased, data-driven methods to model dendritic spine morphology as a continuum rather than a set of isolated categories for advanced studies correlating spine morphology with neuronal function and dysfunction.

A key insight from this study is that the optimal dimensionality reduction method depends on the characteristics of the dataset, particularly imaging resolution and the resulting quality of extracted features. While nonlinear methods such as PCUMAP can better capture complex morphological relationships in high-resolution datasets, they may be more sensitive to noise when feature quality is degraded. In such cases, linear methods like PCA can provide more stable representations. This reinforces the importance of our decision framework, which systematically evaluates dimensionality reduction techniques rather than assuming a single optimal approach. Together, these results demonstrate that dendritic spine morphology is best understood as a continuous, data-dependent structure, where both representation and clustering choices influence biological interpretation. By explicitly evaluating these choices using local, global, and biologically informed metrics, our framework provides a principled approach to studying morphological continua across diverse imaging conditions.

Future work can expand both feature representation and modeling capabilities. In particular, exploring large-scale 3D electron microscopy datasets may enable more detailed quantification of dendritic spine geometry beyond the limits of 2PLSM imaging. Deep learning–based embeddings could also be investigated as a complementary approach for capturing higher-order nonlinear morphological structure directly from image data, potentially identifying features that are difficult to represent using predefined geometric descriptors. However, such approaches typically require substantially larger datasets and introduce variability due to stochastic optimization, which can affect reproducibility and stability of the resulting embedding space. In this study, we intentionally employ deterministic feature-based dimensionality reduction and clustering methods to prioritize interpretability, stability, and reproducibility. Additionally, future work may explore clustering directly in the original high-dimensional feature space and comparing these results with low-dimensional embeddings to assess the robustness of identified morphological structures and the impact of dimensionality reduction on biological interpretation. Extending the framework to incorporate temporal imaging data would further enable investigation of how dendritic spine morphologies evolve over time, allowing dynamic transitions within the morphological continuum to be quantified. The decision framework proposed here can be used in subsequent analyses to determine the most appropriate representation of the spine morphological continuum under different imaging and modeling conditions, supporting more comprehensive data-driven characterization of neuronal structure in development and disease.

## Datasets and data preparation

### Datasets

All imaging, acquisition protocols, and manual annotations described in this section were performed by the original dataset curators.

#### Primary Dataset: Ghani et al.

**Fig 6:**
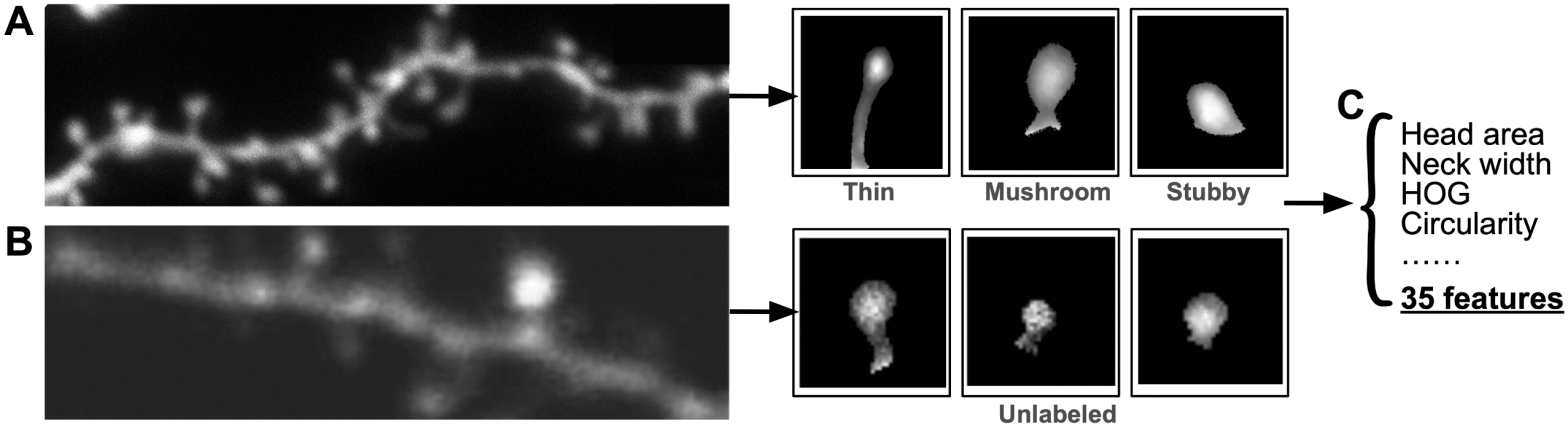
Overview of datasets. **A)** Image sample and expert-identified ROIs (“mushroom”, “stubby”, “thin”) from Ghani et al. **B)** Image sample and identified ROIs (unlabeled) from Smirnov et al. **C)** Shape, contour, and intensity features are collected from both datasets for analysis.

For our primary dataset, we used an open-source, labeled dataset of 456 dendritic spines from postnatal (day 7–10) mouse CA1 hippocampal neurons (Fig 6A) [31]. The hippocampus undergoes rapid structural remodeling during early postnatal development [58], resulting in a wide distribution of developing and intermediate spine morphologies. Images were acquired using two-photon laser scanning microscopy (2PLSM), with Z-stacks collected every 5 minutes over a 4-hour imaging period. Expert annotations provide both spine segmentations and categorical morphological labels (“mushroom”, “stubby”, “thin”), along with manually determined boundaries between the dendrite shaft and individual spines. Although manual spine annotation can exhibit inter-rater variability [24], expert-derived labels remain widely used in the field and provide biologically grounded reference annotations for evaluating computational morphology analysis methods.

The spatial resolution of two-photon microscopy is sufficient to capture subtle morphological changes in dendritic spine geometry, and is therefore a widely used technique for *in vivo* dendritic spine imaging and analysis [59,60]. This makes computational approaches developed using 2PLSM data broadly applicable to commonly used experimental imaging pipelines. In contrast, higher-resolution imaging techniques such as electron microscopy (EM) [17] require extensive sample preparation and specialized acquisition workflows [61], while super-resolution optical methods such as stimulated emission depletion (STED) microscopy can be limited by photobleaching and imaging depth [62,63].

As noted by Ghani *et al.*, the limited axial resolution of 2PLSM motivates the use of two-dimensional maximum-intensity projections (MIPs) derived from Z-stacks. MIPs reduce noise associated with limited depth resolution while preserving the major geometric characteristics of spine morphology [31]. Prior comparative studies further demonstrate that morphometric measurements derived from 2D spine projections are strongly correlated with those obtained from full 3D reconstructions [64]. While absolute measurements such as area or volume may be slightly underestimated in 2D projections, the overall structure of morphological variation and separability of spine classes remain statistically comparable to those observed in 3D datasets [64].

An inherent limitation of using 2D images for dendritic spines is the projection-induced variance due to differing spine attachment angles. However, prior work demonstrates that such variation does not fundamentally distort the morphological relationships captured in 2D datasets [64]. Ghani et al. also utilize consistent imaging geometry and manual verification of segmentations by neuroscientists to help mitigate these concerns.

#### Secondary Dataset: Smirnov et al.

To evaluate the generalizability of our computational framework across datasets, we additionally analyzed a publicly available dataset of labeled dendritic spines from Smirnov *et al.* [32] (Fig 6B). These images were also acquired using two-photon laser scanning microscopy (2PLSM) and capture dendritic spine morphology in hippocampal neurons, and includes manually annotated spine regions. However, no expert annotations were provided in this dataset.

The Smirnov dataset consists of individual two-dimensional optical sections extracted from time-lapse imaging sequences. During acquisition, a 5μm Z-stack composed of five optical planes was collected at each imaging position per minute. The released dataset contains the resulting two-dimensional images rather than full volumetric stacks. Because repeated acquisitions of the same dendritic segments appear within the Smirnov dataset, we implemented a preprocessing procedure to identify and remove duplicate spine observations. Bounding boxes corresponding to the same physical spine were grouped based on spatial proximity and coordinate annotations, and a single representative crop was retained for each spine. This preprocessing step ensures that each spine contributes only one observation to the dataset to preserve the morphological diversity in the original data.

The differences in projection method, spatial resolution, and acquisition protocols are detailed in Table 5. Because our computational pipeline operates on two-dimensional shape representations, these datasets provide a useful opportunity to evaluate the robustness of the learned morphological embeddings across variations in imaging conditions and data collection procedures, as well as how the decision framework may differ across imaging conditions.

**Table 5:** Comparison of imaging scale parameters for the two dendritic spine datasets used in this study.

Parameter	Ghani et al. [31]	Smirnov et al. [32]
Imaging modality	2PLSM	2PLSM
Image size (pixels)	1024 × 1024	128 × 128
Field of view (μm)	19.8 × 19.8	8 × 8 or 16 × 16
Z-stack spacing (μm)	0.3	1.0
Projection	MIP	Single optical plane

### Data preparation

#### Shape, appearance, and intensity-based feature collection.

All preprocessing, feature extraction, and statistical analyses described in this section were performed by the authors using the publicly released datasets. All spines identified by the original dataset publishers were analyzed to limit bias toward spines that are easier to detect computationally.

Shape-based morphological features are fundamental for understanding dendritic spine mechanics, since spine shape is closely linked to synaptic function and plasticity. Existing work to understand dendritic spine shape dynamics points to the actin cytoskeleton and glutamate receptors undergoing rapid and activity-dependent remodeling [34]. Quantitative analysis of these shapes can provide further insight into the mechanisms of synaptic transmission and plasticity. Semi-automatic shape feature collection was performed using SpineJ [35], an ImageJ plugin designed to collect morphological data from high resolution images of the dendritic spine. Because spine ROIs were defined by expert annotations in the original dataset rather than automatically detected by our pipeline, feature extraction was performed on the full set of labeled spines and does not introduce bias toward spines that are easier to detect using automated methods. Details of SpineJ feature collection pipeline can be found in S1 Fig in S1 File.

Intensity features provide complementary information to shape descriptors, especially in 2PLSM images, where intensity patterns can reflect underlying morphological characteristics. Dendritic spine necks typically exhibit a decreased or varied intensity pattern, which may be useful for discriminating spine types with smaller necks that are otherwise difficult to segment based solely on shape [31]. Ghani *et. al* use Histogram of Oriented Gradients (HOG), a method which computes the direction of gradients as an indicator of spine appearance [31]. Another study uses learned intensity-based data from 2PLSM dendritic spine images for segmentation of dendritic spines [16]. Thus, intensity-based descriptors are valuable both for automated segmentation and for capturing morphological heterogeneity not fully explained by geometric features alone.

Contour-based features, including curvature metrics and convex hull ratios, allow further refinement in characterizing dendritic spine morphology. Curvature analysis can highlight regions of high geometric complexity, such as the transition between spine head and neck, or subtle irregularities along the spine perimeter. The convex hull ratio (CHR) quantifies how closely the spine contour approximates a convex shape, which can be indicative of specific spine subtypes or developmental stages [21]. We captured these additional features using a custom automated feature collection pipeline in Python through scikit-learn [65] and OpenCV [66]. All features are listed in S2 Fig in S1 File.

#### C. Feature verification.

Although the feature selection process is supported by existing literature on dendritic spine analysis [10,15,21], to ensure that the features selected were actually representative of meaningful spine variations, we conducted a verification of our selected features using a pairwise comparison between spine types for each feature using nonparametric Mann-Whitney U tests (with *p* < 0.05 for statistical significance) and trained a Random Forest (RF) classifier and assessed feature importance to identify features with low discriminative power for spine type. Details of this analysis can be found in S2 Table and S3 Fig in S1 File. To ensure that feature selection did not overlook multivariate structure, we extended this analysis with additional multivariate evaluations (S5 Fig in S1 File). Specifically, we examined global and class-specific feature–feature correlations to assess potential interaction effects, and performed nested cross-validated forward and backward feature ablation to evaluate redundancy and sufficiency of the selected descriptors. Classification performance plateaued after inclusion of a subset of features, indicating partial redundancy among geometric descriptors while preserving most of the discriminative signal. These analyses led to the 35-dimensional feature set being narrowed down to 31 features, which provides a comprehensive morphometric representation while demonstrating that the retained descriptors are sufficient under cross-validation (S9 Fig in S1 File).

## Supporting information

S1 File Supporting information. Contains S1–S12 Figs and S1–S17 Tables.
(PDF)
