# Correction: A data-driven framework for modeling the dendritic spine continuum using dimensionality reduction and clustering toward understanding synaptic plasticity

**DOI:** 10.1371/journal.pone.0358844

**Published:** 2026-09-18

**Authors:** Uma Shashi Sharma, Philip R. LeDuc, Yongjie Jessica Zhang

The Funding statement for this article is incorrect. The correct Funding statement is as follows: This work was supported by the Carnegie Mellon University Mechanical Engineering department, the National Institutes of Health (R01AG06100501A1), and the US National Science Foundation (CMMI-1953323 and CBET-2332084).

The first author, Uma Shashi Sharma, is incorrectly noted as the corresponding author. The correct corresponding authors are Philip R. LeDuc and Yongjie Jessica Zhang. Philip R. LeDuc’s email address is prl@andrew.cmu.edu and Yongjie Jessica Zhang’s email address is jessicaz@andrew.cmu.edu.
