# Supplementary material for: A data-driven framework for modeling the dendritic spine continuum using dimensionality reduction and clustering toward understanding synaptic plasticity
Source: PLoS One. 2026 Jun 2;21(6):e0349775. doi: 10.1371/journal.pone.0349775 (PMC13229348; doi:10.1371/journal.pone.0349775)
Supplement: S1 File — Contains S1–S12 Figs and S1–S17 Tables. (PDF) [file pone.0349775.s001.pdf]

A Data-Driven Framework for Modeling the Dendritic Spine Continuum Using Dimensionality Reduction and Clustering Toward Understanding Synaptic Plasticity

Uma Shashi Sharma, Philip R. LeDuc, Yongjie Jessica Zhang

Supporting Information

Tables and Figures

Table S1. Summary of related work in dendritic spine dimensionality reduction and clustering, and the methodological gaps addressed in this study

To address the limitations of subjective classification of dendritic spines and work towards advanced models of dendritic spine morphology, researchers have increasingly turned to computational approaches. These computational approaches can be broken into two sub-categories (although some studies employ both): dimensionality reduction, and classification/clustering. Table 1 summarizes the key literature in the field, and specifies the gaps in existing research that our methods fill.

Study	Goal	Imaging technique	Dimensionality reduction (DR) method(s)	Classification ***(CLA)*** / clustering ***(CLU)*** method(s)	Limitations	Gap filled by our work
Rodriguez et al. (2008)	Automated detection, shape analysis, and classification of dendritic spines into 3 categories- higher accuracy and speed than existing technologies	Laser scanning microscopy (LSM)	x	***(CLA)*** Rayburst sampling and pre-existing morphological criteria	Classification method relies on predefined ranges of measurements for general shape groups (stubby, mushroom, thin)	Uses computational modeling methods (DR, clustering) for feature-driven correlation-based clustering, not reliant on predefined morphological groups
Shi et al. (2014)	Semi-supervised approach for morphological classification of dendritic spines into 3 categories (stubby, mushroom, thin spines)	2-photon laser scanning microscopy (2PLSM)	x	***(CLA)*** Semi-supervised learning (SSL) using affinity matrices	Dependence on expert-labeled data, limited to discrete categories	Use expert-labeled data as supplemental information that does *not* directly influence the clustering result (unsupervised clustering)
Ghani et al. (2016)	First implementation of dendritic spine clustering, aiming to confirm known patterns of spine shape and discover new intermediate spine types	2PLSM	Maximum information compression index	***(CLU)*** x-means	Reduced feature set still has 100 features, could lead to data sparsity	Compares multiple DR methods quantitatively across metrics, biological relevance of clustering result is quantitatively analyzed
Ghani et al. (2016)	Comparison of manifold learning techniques and unsupervised machine learning methods for classifying spine types	2PLSM	Principal component analysis (PCA), multidimensional scaling (MDS), Locally linear embedding (LLE), ISOMAP, Local tangent space	***(CLA)*** Support vector machine (SVM), k-nearest neighbors (KNN), random forest (RF)	Found nonlinear dimensionality reduction yields better representation of spine shape than linear methods, but focused on discrete classification	Utilizes more recent advances in nonlinear dimensionality reduction (t-SNE, UMAP) toward a feature-driven quantitative comparative study of dimensionality reduction for

			alignment (LTSA), Laplacian			structure preservation
Ghani et al. (2017)	Automated method for spine shape classification into 3 categories (stubby, mushroom, thin) using shape and appearance features on 2PLSM images	2PLSM	Correlation-based feature selection (CFS), Information gain-based feature selection (IG)	***(CLA)*** Kernel density estimation (KDE), SVM, Neural Network (NN)	Collects high-dimensional feature data but relies on classification into discrete categories	Use clustering technique to represent transitional shape/ morphological continuum
Bokota et al. (2016)	Automatic statistically based computational model of spine shape transitions, comparing group stimulated with LTP, and resting state (control)	Confocal microscopy	PCA	***(CLU)*** cmeans, average-linkage hierarchical	Uses PCA for dimensionality reduction; assumption of linearity and loss of information	Compares multiple DR methods quantitatively across metrics, uses known spine labels as supplemental biological context
Luengo-Sanchez et al. (2018)	Model-based clustering of 3D reconstructed dendritic spines from human cortical pyramidal neurons into 6 categories	Confocal fluorescence microscopy	Multidimensional scaling (MDS)	***(CLU)*** Gaussian mixture model (GMM)	Lacks a comparative study of multiple spine representation and clustering methods and transitional modeling	Inclusion of manual labels and comparison of multiple DR/clustering methods -> more biologically meaningful transitional model of shapes
Kashiwagi et al. (2019)	DR/ ML used for identification of spine types caused by genetic mutations in signal transduction molecules to detect plasticity-related changes in spine head curvature	3D Structured illumination microscopy (SIM)	PCA	***(CLA)*** SVM	PCA assumes linearity; could introduce potential clustering bias. Classification of spines into discrete categories (mushroom, non-mushroom)	Combines nonlinear DR with clustering approaches, manual labels do not influence cluster assignment (data-driven)
Ofer et al. (2022)	Analyzed morphological features of human and mouse spines, found evidence of shape continuum and structural differences across species	Confocal microscopy	x	No classification or clustering; analyzed morphological parameter distributions	Identified morphological continuum based on simple feature set (head volume, neck length + diameter), didn't consider feature correlations	Advanced morphological continuum representation using feature correlations across shape, intensity, and contour information
Choi et al.	Visualization	SIM	PCA, t-SNE,	***(CLA)***	Qualitative survey	Includes

(2022)	framework for an interactive dendritic spine analysis, focuses on user experience with interactive clustering and analysis of spines		UMAP (for visualization)	machine-learning enabled classification (for visualization)	of experts found that tSNE was the preferred visualization method, but no quantitative analysis to support this finding	quantitative analysis of DR methods based on local and global structural preservation
Argunşah et al. (2022)	Automated detection, segmentation, and spine neck clustering graphical interface.	2PLSM and confocal laser scanning microscopy (CLSM)	x	***(CLA)*** Convolutional Neural Network w/ SURF (CNN)	Morphometric quantification of features (spine volume, neck length, etc) but does not apply cluster analysis or dimensionality reduction	Utilizes high-level feature information and unsupervised clustering for representation of spine morphometry
Pchitskaya et al. (2023)	End-to-end pipeline for image segmentation, spine geometry feature extraction, spine classification/ clustering for 3D images	Confocal microscopy	PCA	***(CLA)*** SVM, ***(CLU)*** k-means	Reliance on linear dimensionality reduction and hard clustering methods that could obscure relationships in data, limited comparative study	Includes quantitative analysis of DR methods based on local and global structural preservation, probabilistic clustering to model transitional shapes
Fernholz et al. (2024)	Fully automated deep-learning based method to quantify dendritic spines in microscopy	Two-photon microscopy	x	***(CLA)*** DBSCAN, k-means	Focuses on segmentation accuracy and alignment of classification results to human-expert labels and annotations, but no comparative analysis	Application of non-linear dimensionality reduction toward unsupervised clustering rather than classification

Figure S1. Morphological Feature Collection Pipeline using SpineJ.

A maximum intensity projection (MIP) image is loaded into ImageJ before launching the SpineJ plugin. Once the plugin is launched, the user is prompted to control segmentation parameters to ensure enough smoothing while still retaining shape properly. The image is binarized, and the user is able to manually redefine spine necks that were not captured in the segmentation. The plugin automatically identifies spines in the image, and the user is able to manually define the neck-head junction.

The SpineJ software yields 15 total shape-based features: Neck length, Spine Length, Ratio (%), Minor axis, Major axis, Aspect Ratio, Perimeter head, Area head, Smallest neck width, Median neck width, Average neck width, Mean FWHM, Std FWHM, Mean neck fit goodness, Standard deviation neck fit goodness.

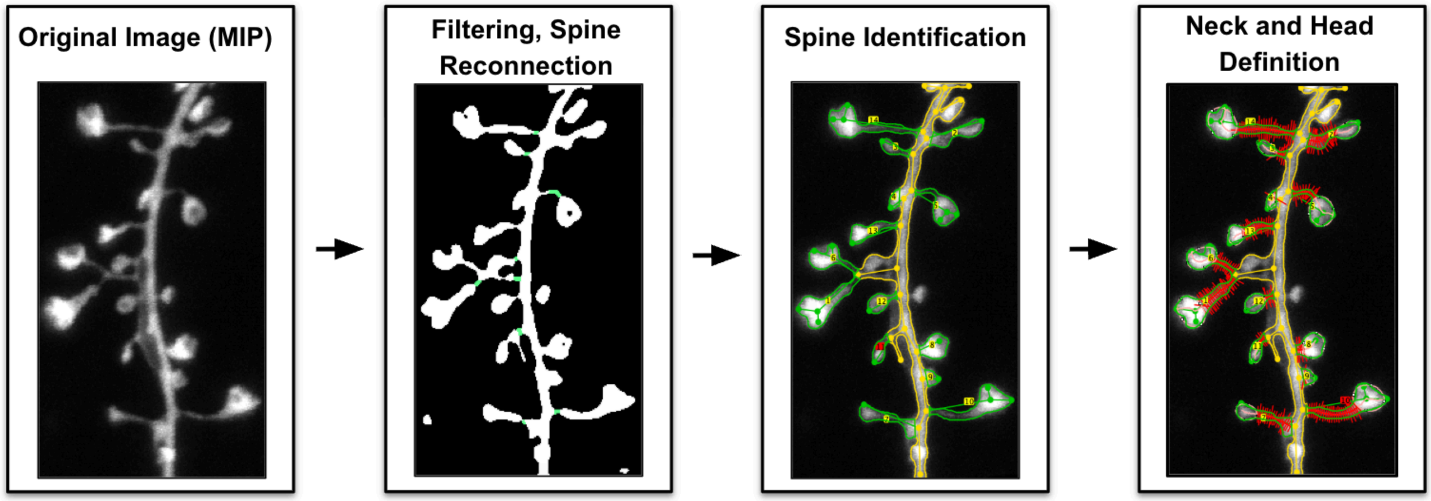

Figure S2. Morphological parameter distributions: the distributions of each of the 32 shape, contour, and intensity based features across mushroom, stubby, and thin spine types.

Mean and standard deviation values for each feature across each group of spine types, which gives a visual representation of which features are similar vs. highly discriminative across spine types.

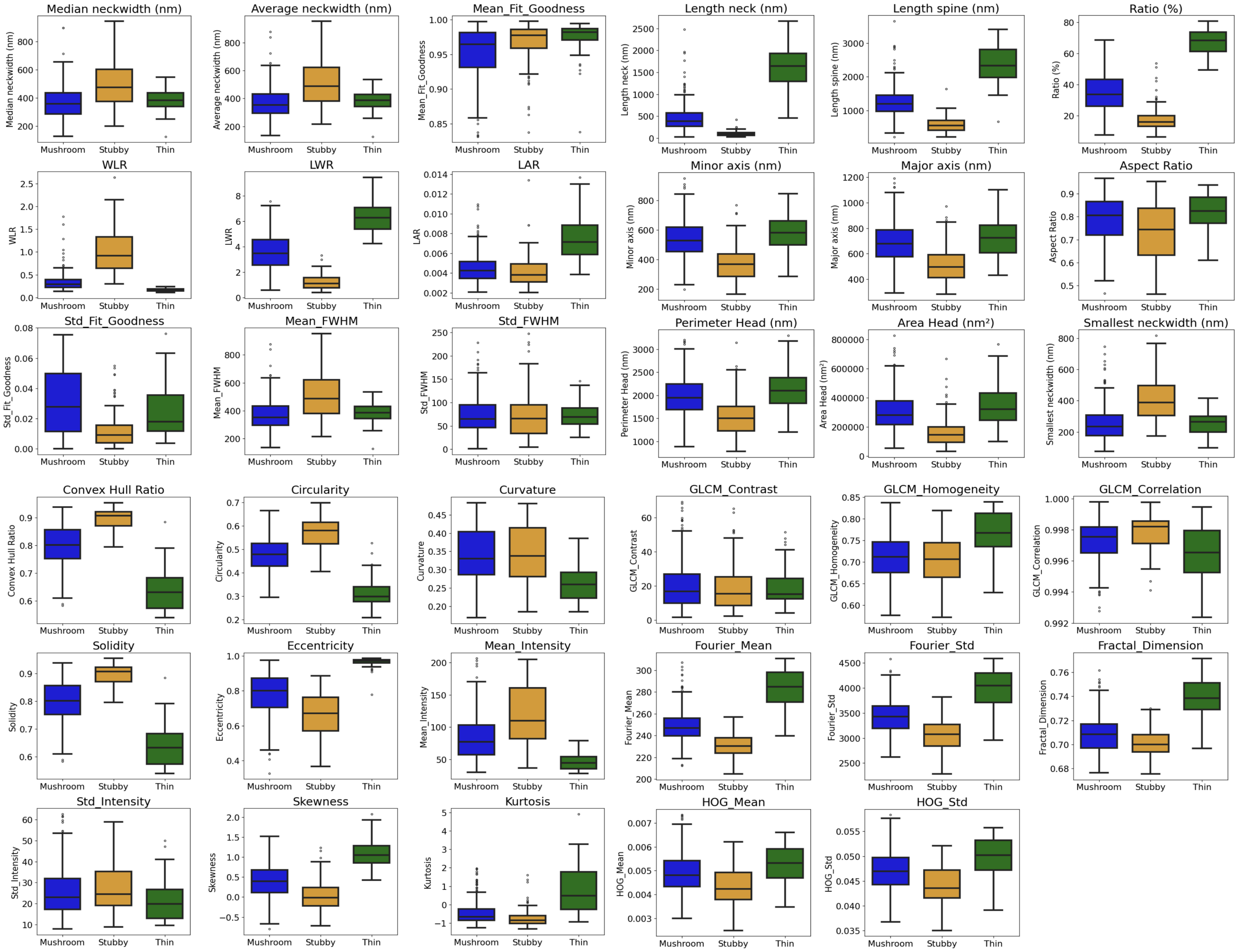

Table S2. Pairwise p-values for spine feature comparison using Mann-Whitney U test across mushroom, stubby, and thin spine types.

Although the feature selection process is backed up by existing literature on dendritic spine analysis, we wanted to ensure that the features selected were actually representative of meaningful spine variations. This means that in general, despite the continuum of spine morphology, we should observe trends for spine shape within each of the manual clusterings. If there are features that show no discrimination across all three groups, they may not represent valuable information about variability across spines.

For this reason, we conducted verification of our selected features in two steps. First, we conducted a pairwise p-value test between spine types for each feature using non-parametric Mann–Whitney U tests. if $p < 0.05$, there is a statistically significant difference for this feature between groups. If $p >> 0.05$, there is no statistically significant difference between the two groups.

We use $p < 0.05$ as our primary threshold for identifying statistically significant differences in feature distributions across spine types, but since spine morphology lies on a continuum some degree of overlap between groups is expected. Therefore, for features with marginal p-values ($0.05 < p < 0.1$), we considered retaining them if they showed relevance in machine learning-based importance rankings, to balance statistical insights with biological interpretability.

	**Mushroom vs. Stubby**	**Mushroom vs. Thin**	**Stubby vs. Thin**
**Length neck (nm)**	0.0	0.0	0.0
**Length spine (nm)**	0.0	0.0	0.0
**Ratio (%)**	0.0	0.0	0.0
**Minor axis (nm)**	0.0	0.034	0.0
**Major axis (nm)**	0.0	0.205	0.0
**Aspect ratio**	0.0	0.088	0.0
**Perimeter (nm)**	0.0	0.071	0.0
**Area Head (nm^2)**	0.0	0.089	0.0
**Smallest neck width (nm)**	0.0	0.354	0.0
**Median neck width (nm)**	0.0	0.047	0.0
**Average neck width (nm)**	0.0	0.066	0.0
**Mean fit goodness**	0.0	0.0	0.258
**Width-to-length ratio (WLR)**	0.0	0.0	0.0
**Length-to-width ratio (LWR)**	0.0	0.0	0.0
**Length-to-area ratio (LAR)**	0.012	0.0	0.0
**Std. fit goodness**	0.0	0.105	0.0
**Mean FWHM**	0.0	0.067	0.0
**Std. FWHM**	0.537	0.367	0.248
**Convex hull ratio**	0.0	0.0	0.0

**Circularity**	0.0	0.0	0.0
**Curvature**	0.337	0.0	0.0
**Solidity**	0.0	0.0	0.0
**Eccentricity**	0.0	0.0	0.0
**Mean Intensity**	0.0	0.0	0.0
**Std. Intensity**	0.161	0.001	0.0
**Skewness**	0.0	0.0	0.0
**Kurtosis**	0.0	0.0	0.0
**GLCM Contrast**	0.433	0.766	0.750
**GLCM Homogeneity**	0.135	0.0	0.0
**GLCM Correlation**	0.0	0.0	0.0
**Fourier mean**	0.0	0.0	0.0
**Fourier std.**	0.0	0.0	0.0
**Fractal dimension**	0.0	0.0	0.0
**HOG mean**	0.0	0.017	0.0
**HOG std.**	0.0	0.0	0.0

Figure S3. Classification based on manual labels and feature importances

The second step for feature verification was to analyze feature importances based on spine classification. We conducted this analysis by implementing a Random Forest (RF) classifier and analyzing feature importances that contributed to accurate classification of spine types into the manual labels. We implemented the RF classifier using Synthetic Minority Oversampling Technique (SMOTE) to account for the over-representation of mushroom spines in the dataset, standardized morphometric features, and obtained optimal hyperparameters (n_estimators, max_depth, max_features, min_samples_split, min_samples_leaf) using 5-fold cross-validation grid search. An accuracy of 94% was reached, and the confusion matrix is shown in A. Some examples of misclassified spines are shown in B.

The features with low importance toward classification (C), in conjunction with parameter distributions and p-values from Figure S2 and Table S2 resulted in 4 features being dropped from the dataset: Standard deviation FWHM, Mean fit goodness, GLCM Contrast, and GLCM Correlation.

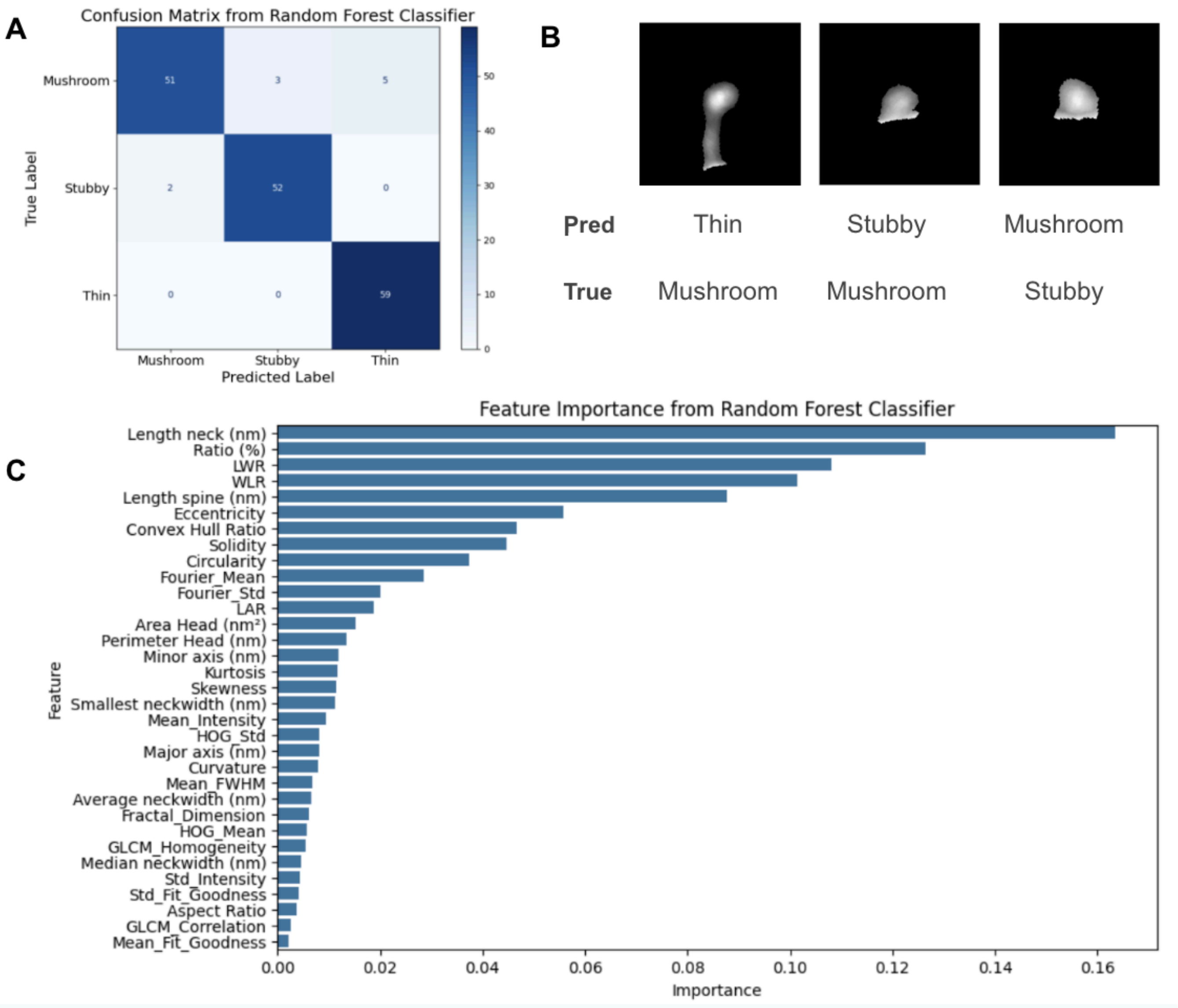

Figure S4. Loadings obtained from PCA projection and most important features contributing to each principal component (PC).

The principal components obtained from PCA tell us the directions of highest variation in the data, determined based on a weighted linear combination of the high-dimensional feature set. The loading scores for each principal component are these weights, and analyzing the highest-weighted features for each principal component gives insight into the axes of highest variation in the dataset, indicative of which morphological features are most influential to spine morphology. The top 5 features for each principal component are detailed in A. PC1, which represents the majority of the variance in the data, is dominated by neck and spine length, which makes sense since these features are commonly used to discriminate spine types.

A

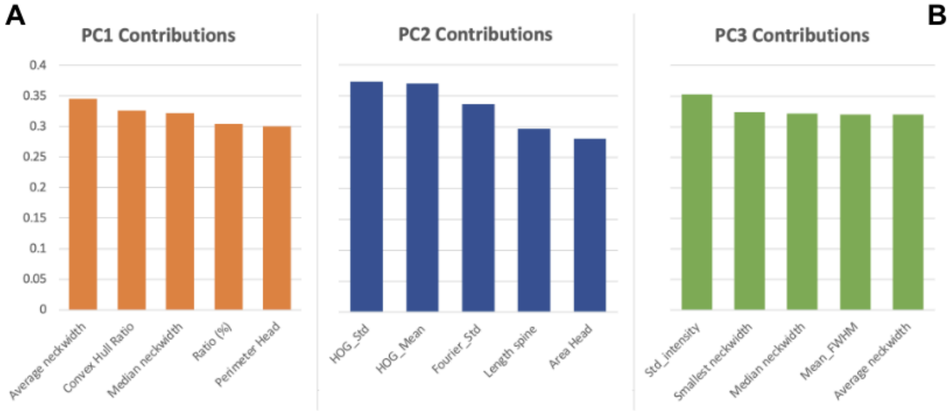

B

	**Feature contributions**
**PC1**	Neck length, spine length
**PC2**	Head/neck ratio, Convex hull ratio
**PC3**	Intensity, neck width

Figure S5. Multivariate feature structure and contribution of morphological descriptors

The goal of this analysis step is to determine if the feature data contains any multivariate structure. This analysis was done in three phases. (A) We generated a Spearman correlation heatmap of all extracted spine features. Strong intra-group correlations are observed among geometric size metrics (spine length, major/minor axis, area, perimeter) and among intensity/texture descriptors (skewness, kurtosis, Fourier features, HOG statistics), while several cross-group relationships remain weak to moderate. This indicates partial redundancy of additional features, but preserved complementary information across feature categories. (B) Nested cross-validation performance across feature subsets (mean ± SD balanced accuracy). Models trained on geometric features alone capture substantial discriminatory structure; however, inclusion of the full feature set yields comparable or modestly improved performance, indicating that non-geometric descriptors may contribute complementary information. (C) Nested cross-validation forward feature ablation. Predictive performance increases rapidly with the first several ranked features and then stabilizes, demonstrating diminishing marginal gains in classification accuracy. However, performance remains stable or slightly improved with inclusion of additional features, indicating that the broader feature set does not degrade model performance and may preserve subtle multivariate structure not captured by minimal subsets. Together, these analyses demonstrate that although a compact subset of features captures coarse morphological variation, the full multivariate feature set preserves complementary geometric, curvature, and texture information. This supports the use of the larger feature space, rather than just selecting few features, for unsupervised clustering, where capturing transitional and high-dimensional morphological structure is critical.

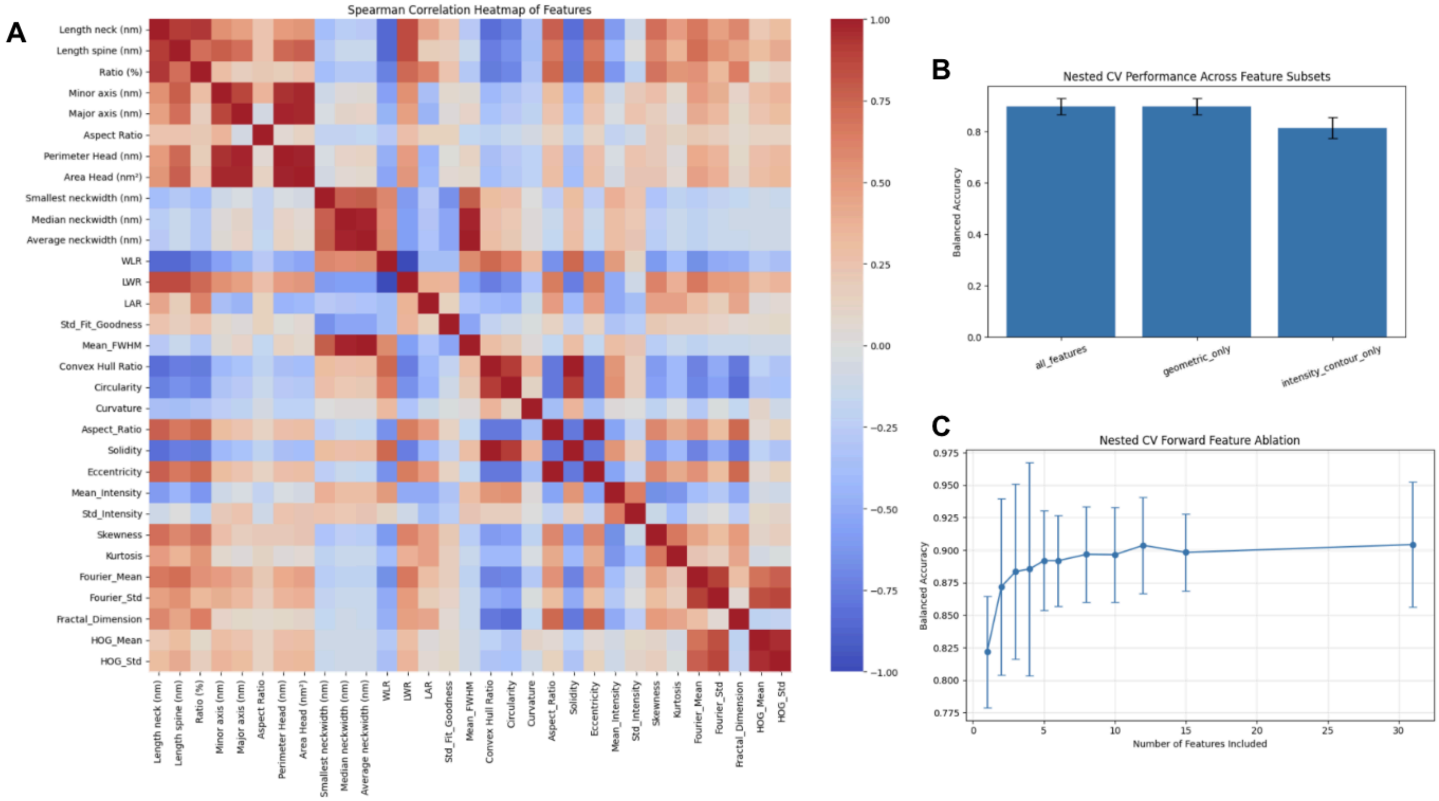

Table S3. Embedding Dimensionality Sensitivity Analysis (2–5 Dimensions) Across Dimensionality Reduction Methods

We evaluated embedding dimensionality from 2–5 dimensions across all DR methods. Increasing dimensionality improved geometric preservation (trustworthiness and distance correlations) for all methods; however, clustering quality did not monotonically improve. For PCUMAP, silhouette and Xie–Beni indices peaked at 3 dimensions and remained stable thereafter, indicating that higher-dimensional embeddings did not yield additional biologically meaningful structure. In contrast, PCA showed improved distance preservation at higher dimensions but progressively degraded cluster compactness, showing that geometric fidelity does not guarantee interpretable clustering by itself.

Method	Dim	T(k)	PearsonDist	Spearman Dist	Avg Label Overlap	Silhouette (k=5)	CH (k=5)	DB (k=5)	Xie–Beni (k=5)	Avg Max Prob	Norm Entropy
**ISOMAP**	2	0.8939	0.9161	0.8938	0.4827	0.3702	405.20	0.8403	0.1686	0.6885	0.5457
**ISOMAP**	3	0.9368	0.9350	0.9208	0.4917	0.3020	293.59	1.0452	0.3242	0.6039	0.6601
**ISOMAP**	4	0.9660	0.9484	0.9394	0.4917	0.2588	229.64	1.2194	0.5983	0.5405	0.7344
**ISOMAP**	5	0.9708	0.9544	0.9466	0.4917	0.2375	200.80	1.3030	0.8700	0.5085	0.7688
**PCA**	2	0.8791	0.9046	0.8899	0.4161	0.3739	461.76	0.8334	0.2234	0.6951	0.5345
**PCA**	3	0.9369	0.9612	0.9494	0.4884	0.2879	263.26	1.0821	0.4560	0.5961	0.6633
**PCA**	4	0.9720	0.9782	0.9760	0.4884	0.2078	184.57	1.3868	0.9606	0.5116	0.7563
**PCA**	5	0.9826	0.9877	0.9845	0.4884	0.1775	155.19	1.5094	1.4373	0.4691	0.8024
**PCUMAP**	2	0.9192	0.7016	0.8321	0.3254	0.2111	48.08	1.3322	2.4104	0.7001	0.4970
**PCUMAP**	3	0.9600	0.9264	0.9144	0.3016	0.3553	381.89	0.9403	0.2394	0.6476	0.6015
**PCUMAP**	4	0.9723	0.9352	0.9246	0.2695	0.3386	341.16	0.9978	0.3004	0.6250	0.6323
**PCUMAP**	5	0.9737	0.9365	0.9263	0.3150	0.3316	333.63	1.0171	0.3122	0.6192	0.6391
**t-SNE**	2	0.9558	0.7689	0.7583	0.4067	0.4091	594.52	0.7950	0.1192	0.7038	0.5298
**t-SNE**	3	0.9754	0.7982	0.7903	0.4788	0.3421	403.09	1.0139	0.1862	0.6331	0.6364
**t-SNE**	4	0.9823	0.8393	0.8405	0.4675	0.2868	284.40	1.1907	0.2887	0.5707	0.7102
**t-SNE**	5	0.9846	0.8166	0.8150	0.5636	0.2036	170.11	1.5306	2.0690	0.4436	0.8365
**UMAP**	2	0.9272	0.7873	0.7824	0.1970	0.4009	600.57	0.8238	0.1276	0.7022	0.5307
**UMAP**	3	0.9544	0.8232	0.8225	0.3456	0.3558	440.83	0.9799	0.2097	0.6481	0.6105

**UMAP**	4	0.9667	0.8321	0.8301	0.2829	0.3401	422.40	1.0308	0.2555	0.6304	0.6327
**UMAP**	5	0.9692	0.8307	0.8283	0.4434	0.3412	420.30	1.0368	0.2683	0.6287	0.6352

Figure S6. Multi-angle views of dimensionality reduction result from the dendritic spine feature dataset using 5 dimensionality reduction methods (PCA, ISOMAP, tSNE, UMAP, PCUMAP)

Viewing the dimensionality reduction results can be valuable for multiple reasons. These additional angles can reveal hidden cluster structure and provide orthogonal perspectives that demonstrate the primary axes of variation. The multi-angle view shows us that t-SNE still has the greatest data spread, while the PCUMAP projection is the most compact across all views.

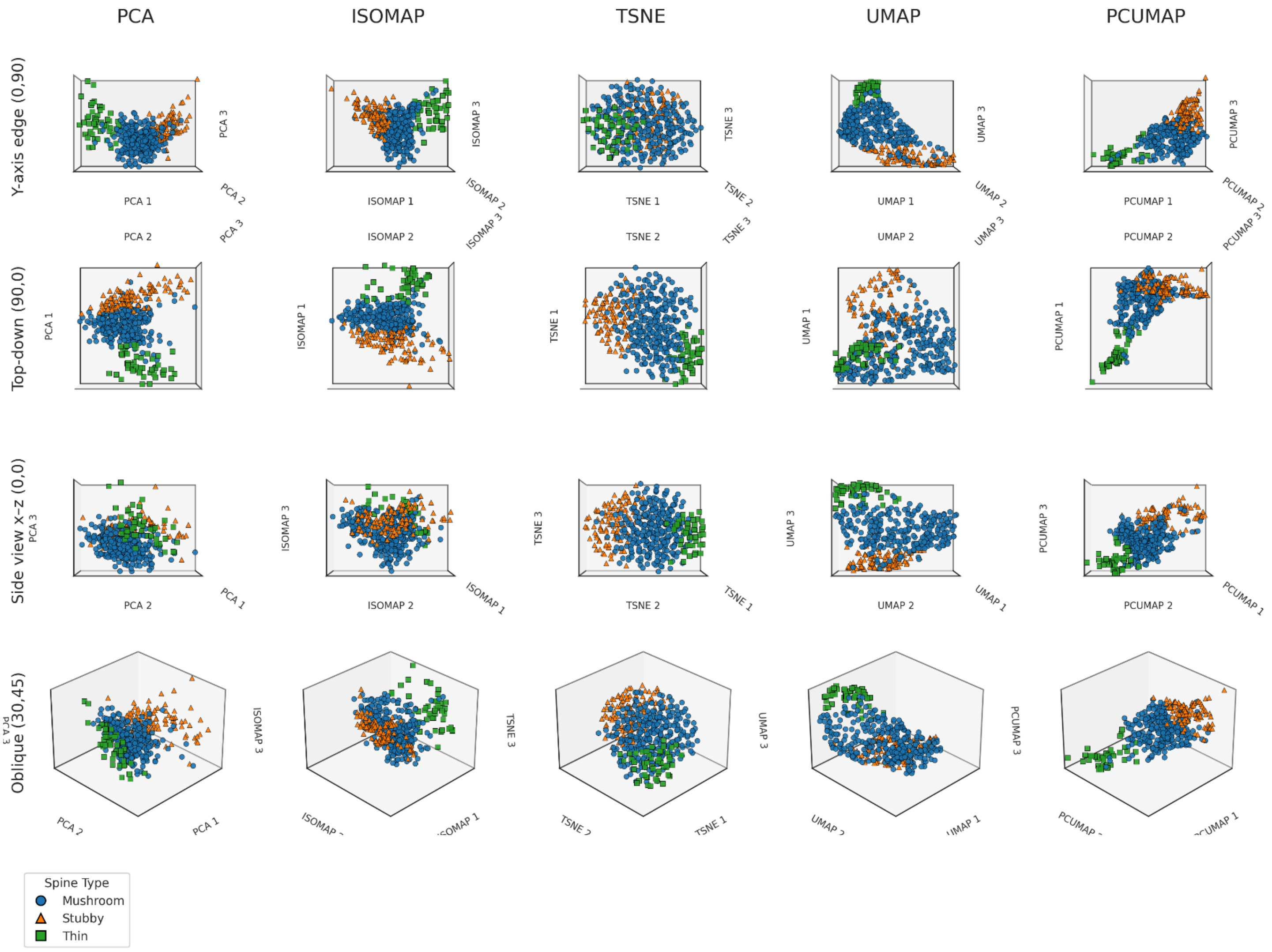

Table S4. Hyperparameters used for dimensionality reduction methods presented in main figures

Method	Hyperparameter	Value
**PCA**	Number of components (n_components)	3
	Other parameters	Default (scikit-learn)
**ISOMAP**	Number of components (n_components)	3
	Number of neighbors (n_neighbors)	30
**t-SNE**	Number of components (n_components)	3
	Perplexity	100
	Random seed (random_state)	1212
**UMAP**	Number of components (n_components)	3
	Number of neighbors (n_neighbors)	30
	Random seed (random_state)	42
**PCUMAP**	Number of components (n_components)	3
	Minimum distance (min_dist)	0.3
	Correlation loss weight	1000
	Constraint strength (beta)	10
	Random seed	1178

Table S5. Hyperparameter selection for PCUMAP

In PCUMAP, correlation loss weight defines how strongly PCUMAP penalizes deviations from the original correlation matrix of the high-dimensional data, while beta controls how much PCUMAP prioritizes correlation preservation vs. local structure. From our experimentation, we found that increasing correlation loss weight and beta resulted in an increase of global structure preservation, while still keeping local structure preservation consistent.

**Correlation loss weight**	**Beta**	**Spearman correlation**	**Pearson correlation**
**100**	5	0.850	0.848
**200**	5	0.866	0.864
**200**	10	0.866	0.864
**300**	10	0.877	0.874
**500**	10	0.896	0.890
**1000**	10	0.926	0.914

Table S6. Sensitivity analysis for number of nearest neighbors for calculation of Trustworthiness and Local Continuity Meta Criterion (T(k) and LCMC)

Trustworthiness (T(k)) and Local Continuity Meta Criterion (LCMC), $\in [0, 1]$ are two metrics that were used to evaluate local structure preservation of dimensionality reduction techniques. Both metrics depend on a hyperparameter *k,* or the number of nearest neighbors to a given point that are considered in the calculation. We performed a sensitivity analysis, varying *k* within the range $5 \leq k \leq 50$, to evaluate the robustness of our methods selection protocol to variations in the number of neighbors. Typically, a low number of neighbors (e.g. $k = 5$) may be more sensitive to noise, while a high number of neighbors (e.g. $k = 50$) may miss details.

The results of this sensitivity analysis show that t-SNE achieves the highest local structure preservation across all values of *k*, while PCUMAP consistently ranks second. Additionally, all methods demonstrate stable performance across the scales tested, supporting robustness of the dimensionality reduction methods across hyperparameter values.

**k**	**PCA**		**ISOMAP**		**t-SNE**		**UMAP**		**PCUMAP**	
	**T(k)**	**LCMC**	**T(k)**	**LCMC**	**T(k)**	**LCMC**	**T(k)**	**LCMC**	**T(k)**	**LCMC**
**5**	0.935	0.444	0.928	0.435	**0.976**	**0.576**	0.962	0.543	0.965	0.538
**10**	0.935	0.444	0.928	0.435	**0.975**	**0.549**	0.962	0.543	0.966	0.545
**25**	0.945	0.562	0.930	0.520	**0.964**	**0.630**	0.944	0.560	0.953	0.576
**50**	0.953	0.677	0.935	0.616	**0.958**	**0.694**	0.937	0.644	0.948	0.650

* **best method**, second best method

Table S7. Sensitivity analysis for computation of Structure Preservation Score (SPS) from Local Score (LS) and Global Score (GS).

In the Methods, we defined a Structure Preservation Score that is defined as the weighted average of the Local Score (LS) and Global Score (GS), and is used to aid in the determination of dimensionality reduction method for spine morphology representation that is best able to balance between local and global structure preservation. This equation is defined as: **$SPS = LS * w_{LS} + GS * w_{GS}$**

The results presented in the manuscript defined w_{LS}=0.5 and w_{GS}=0.5, giving equal preference to local and global structure preservation, resulting in the selection of PCUMAP for dimensionality reduction. This table presents a sensitivity analysis examining the quantitative result of adjusting these weight parameters, where the best method is underlined and bolded and the second best result is bolded. In practice, researchers may want to prioritize preserving local or global structure depending on if their later analysis is more reliant on broad, large-scale structural accuracy or fine-grained transitional accuracy in the low-dimensional embedding.

The results from tuning these parameters show that PCUMAP consistently performed the highest among the 5 tested dimensionality reduction methods, even when varying the weights in the SPS calculation. PCA performs the second best in most cases. When LS is heavily preferred over GS (0.7/0.3), UMAP and PCUMAP become the best performing methods, which makes sense since these projections are meant to preserve neighborhood relationships. When LS holds lower weight compared to GS (0.3/0.7), PCA has the best performance, although it is closely followed by PCUMAP.

The results of this sensitivity analysis justify the use of PCUMAP as the preferred dimensionality reduction method for our dataset.

	**Weighting scheme (w_{LS}/w_{GS})**		**0.5/0.5**	**0.6/0/4**	**0.7/0.3**	**0.4/0.6**	**0.3/0.7**
**DR method**	**Local Score (LS)**	**Global Score (GS)**	**Structure Preservation Score (SPS)**				
**PCA**	0.690	0.955	0.822	0.796	0.770	0.849	**0.876**
**ISOMAP**	0.682	0.910	0.796	0.723	0.750	0.819	0.841
**t-SNE**	0.762	0.772	0.767	0.766	0.765	0.768	0.769
**UMAP**	0.752	0.823	0.788	0.781	0.774	0.795	0.801
**PCUMAP**	0.753	0.920	**0.838**	**0.820**	**0.802**	**0.853**	0.870

* **best method**, second best method

Table S8. Sensitivity analysis for the weighting scheme of Biological Transition Score (BTS).

The Biological Transition Score (BTS) was developed to use biological insights from dendritic spine literature to understand how well dimensionality reduction methods preserve known functional and developmental relationships between spine types. Many studies have reported general trends about dendritic spine growth and elimination, namely that thin spines either mature into mushroom spines or are eliminated, stubby and thin spines can evolve into mushroom spines during long-term potentiation, and stubby spines retract or shrink during long-term depression. Stubby spines do not commonly evolve directly to thin spines [16, 17].

Because there is no widely accepted quantitative model describing "transition rates" between spine morphologies, the transition weights used in BTS rely on biologically motivated approximations. To evaluate the robustness of this metric to these assumptions, we performed a sensitivity analysis across multiple transition-weighting schemes to evaluate the stability of the results under these different conditions.

The weights are defined in the table below as the following:

- "Same type": Mushroom→Mushroom, Stubby→Stubby, Thin→Thin
- "Common": Mushroom↔Thin, Mushroom↔Stubby
- "Uncommon": Stubby↔Thin

We grouped the tested transition weighting configurations into three biologically interpretable categories based on the emphasis placed on "Same type" consistency, biologically "common" spine type transitions, and penalization of "uncommon" transitions.

1. **Restrictive weighting schemes**: Favor local morphological homogeneity with strict penalties for uncommon transitions, assuming strong biological separation between groups. Colored orange.
2. **Moderate weighting schemes:** Balances within-type preservation with cross-type relationships, still penalizes rare transitions. Reflects more gradual maturation to and from mushroom spines, while uncommon transitions are still not favored. Colored blue.
3. **Non-restrictive weighting schemes:** More relaxed assumptions about morphological separation, allowing greater neighborhood mixing between different spine types for a more continuous morphological landscape while still weighting less common transition types lower. Colored gray.

The results of this sensitivity analysis show that across all tested weighting schemes, UMAP consistently achieves the highest BTS, followed closely by PCUMAP . Absolute BTS values varied when the emphasis was placed on common cross-type transitions, but method rankings remain stable. Additionally, changes to uncommon transition weights had minimal impact, suggesting that BTS is primarily driven by preservation of gradual development toward mushroom type spines, and is not significantly impacted by rare transition types.

**Distance Metric**	**Same type**	**Com mon**	**Unco mmon**	**PCA**	**ISOMAP**	**t-SNE**	**UMAP**	**PCUMAP**
**Euclidean**	1.0	0.5	0.0	0.536	0.421	0.180	**0.670**	0.664
	0.8	0.25	0.0	0.564	0.459	0.602	**0.687**	0.681
	0.8	0.25	0.25	0.564	0.459	0.602	**0.687**	**0.681**
	0.8	0.5	0.0	0.532	0.420	0.573	**0.664**	0.657
	0.8	0.5	0.25	0.532	0.420	0.573	**0.664**	0.657
	0.8	0.75	0.0	0.516	0.393	0.562	**0.663**	0.655
	0.8	0.75	0.25	0.516	0.393	0.562	**0.663**	0.655
	1.0	0.25	0.0	0.571	0.468	0.610	**0.694**	0.689

	1.0	0.25	0.25	0.571	0.468	0.610	**0.694**	0.689
	1.0	0.75	0.0	0.524	0.408	0.567	**0.661**	0.654
	1.0	0.75	0.25	0.524	0.408	0.567	**0.661**	0.654
	1.2	0.25	0.0	0.578	0.578	0.474	0.615	**0.694**
	1.2	0.25	0.25	0.578	0.578	0.474	0.615	**0.694**
	1.2	0.75	0.0	0.551	0.445	0.590	**0.677**	0.671
	1.2	0.75	0.25	0.551	0.445	0.590	**0.677**	0.671
**Cosine**	1.0	0.5	0.0	0.867	0.881	0.882	**0.932**	0.903
	0.8	0.25	0.0	0.900	0.910	0.910	**0.951**	0.927
	0.8	0.25	0.25	0.900	0.910	0.910	**0.951**	0.927
	0.8	0.5	0.0	0.855	0.869	0.870	**0.927**	0.893
	0.8	0.5	0.25	0.855	0.869	0.870	**0.927**	0.893
	0.8	0.75	0.0	0.890	0.898	0.891	**0.967**	0.919
	0.8	0.75	0.25	0.890	0.898	0.891	**0.967**	0.919
	1.0	0.25	0.0	0.913	0.922	0.922	**0.959**	0.937
	1.0	0.25	0.25	0.913	0.922	0.922	**0.959**	0.937
	1.0	0.75	0.0	0.854	0.867	0.866	**0.930**	0.891
	1.0	0.75	0.25	0.854	0.867	0.866	**0.930**	0.891
	1.2	0.25	0.0	0.923	0.931	0.930	**0.965**	0.945
	1.2	0.25	0.25	0.923	0.931	0.930	**0.965**	0.945
	1.2	0.75	0.0	0.855	0.869	0.869	**0.927**	0.893
	1.2	0.75	0.25	0.855	0.869	0.869	**0.927**	0.893

* **best method**, second best meth

Figure S7. Transition matrices showing the Euclidean nearest neighbors of each cluster.

The transition matrices below were used to create Figure 3C in the manuscript. These matrices define the percentage of spine types whose direct nearest neighbor belongs to each other cluster, where "from" denotes the cluster assignment of the current spine and "to" denotes the cluster assignment of the nearest spine. These transition matrices show that FCM and GMM have a higher "overlap" between spines belonging to different clusters, while hierarchical clustering demonstrates more highly separated clusters. The results of this analysis were visualized using the transition graph in Figure 3C for ease of visualization and interpretability.

Hierarchical Clustering

	C1	C2	C3	C4	C5
C1	0.97	0.01	0.00	0.00	0.01
C2	0.01	0.97	0.00	0.00	0.01
C3	0.00	0.02	0.98	0.00	0.00
C4	0.00	0.00	0.00	0.99	0.01
C5	0.01	0.00	0.00	0.01	0.97

FCM Clustering

	C1	C2	C3	C4	C5
C1	0.89	0.05	0.00	0.02	0.04
C2	0.03	0.92	0.02	0.00	0.03
C3	0.00	0.08	0.92	0.00	0.00
C4	0.04	0.00	0.00	0.92	0.04
C5	0.04	0.03	0.00	0.01	0.92

GMM Clustering

	C1	C2	C3	C4	C5
C1	0.94	0.05	0.00	0.01	0.01
C2	0.02	0.90	0.06	0.00	0.02
C3	0.00	0.08	0.92	0.00	0.00
C4	0.01	0.00	0.00	0.95	0.05
C5	0.01	0.01	0.00	0.03	0.95

Table S9. Structural and interpretability metrics comparing K = 3 and K = 5 clustering solutions

To further assess whether the five-cluster solution represents meaningful morphological refinement rather than over-segmentation, we performed a direct comparison between clustering based on (1) the ground-truth expert labels, (2) using K = 3 clusters, corresponding to the canonical "mushroom," "stubby," and "thin" classes, (3) clustering using K = 5, as suggested by the analysis in Figure S6, where K is the number of clusters. These analyses were performed using identical embeddings, clustering algorithms, and evaluation metrics. This way, we can understand improvements in structural resolution using the five-cluster solution compared to both the ground truth labels and three-cluster approach.

We report the same structural and probabilistic metrics described in the main text for both K = 3 and K = 5, including the Silhouette score, Calinski-Harabasz (C-H) score, and Davies-Bouldin (D-B) score, which quantify cluster compactness and separation, as well as Average Entropy, Average Sharpness, and Average Maximum Membership Probability for probabilistic clustering methods. Comparable entropy and membership profiles between K = 3 and K = 5 indicate that the five-cluster solution maintains continuity and probabilistic interpretability while resolving additional substructure. We additionally report label-alignment metrics, including the Adjusted Rand Index (ARI), Normalized Mutual Information (NMI), and cluster purity, which quantify how closely the clusters align with expert annotations. As expected, these metrics favor K = 3 due to the three-class nature of the labels; however, they remain high at K = 5, indicating that the additional clusters may refine existing categories rather than creating implausible groupings. Additionally, K = 3 and K = 5 clustering solutions both achieved equal or stronger structural separation than the expert ground-truth labels evaluated in the same embedding.

Together, these results demonstrate that clustering at K = 5 achieves structural quality comparable to the canonical three-group organization and to the expert labels themselves, while providing increased morphological resolution. This supports our interpretation that five clusters capture biologically meaningful heterogeneity within traditional spine categories, rather than representing over-segmentation driven by noise or model complexity.

**Clustering method**	**Ground Truth**	**Hierarchical**		**FCM**		**GMM**	
**Number of clusters**	**3 (expert annotated)**	**3**	**5**	**3**	**5**	**3**	**5**
**Silhouette Score**	0.308	0.347	0.324	0.380	0.351	0.329	0.281
**C-H Score**	277.328	326.227	336.848	395.729	381.613	337.783	280.850
**D-B Score**	0.934	0.938	0.964	0.901	0.957	0.995	1.047
**Avg Entropy**	–	–	–	5.623	5.397	0.213	0.239
**Avg Sharpness**	–	–	–	0.075	0.112	0.806	0.852
**Avg Max Prob**	–	–	–	0.992	0.970	0.911	0.905
**ARI**	–	0.308	0.199	0.581	0.267	0.366	0.260
**NMI**	–	0.445	0.377	0.571	0.436	0.420	0.424
**Purity**	–	0.727	0.809	0.860	0.827	0.791	0.873

Table S10. Structural validity and stability metrics for K = 2, 5, and 8 clustering solutions on the PCUMAP embedding

Clustering results were evaluated using complimentary validity indices, including Silhouette score (cluster compactness and separation), Davies–Bouldin (cluster overlap; lower is better), and subsampling stability assessed using the Adjusted Rand Index (mean ± standard deviation across resampling runs).

K = 2 represents a coarse representation of spine structure, while K = 8 reflects a higher-resolution subdivision of substructural types. Although separation metrics continued to improve modestly at higher K values, stability and interpretability decreased. K = 5 provided a balanced trade-off between compactness, separation, and clustering stability without evidence of excessive fragmentation.

Method	K	Silhouette	Davies–Bouldin	Stability (ARI mean ± SD)
**FCM**	2	0.365	1.056	0.962 ± 0.032
**FCM**	5	0.316	0.941	0.845 ± 0.086
**FCM**	8	0.288	0.889	0.792 ± 0.095
**GMM**	2	0.316	1.142	0.880 ± 0.070
**GMM**	5	0.311	1.086	0.606 ± 0.186
**GMM**	8	0.289	0.969	0.644 ± 0.103

Table S11 Cluster size (K=2, 5, 8) balance and probabilistic interpretability metrics

In order to see how various sizes, including a coarse representation (K=2) and a subdivided representation (k=8) impact interpretability, we conducted multiple cluster size diagnostic studies. This includes analyzing the largest and smallest cluster fractions and the standard deviation of cluster sizes, which was used to quantify partition balance. We also used the average maximum membership probability (confidence), normalized entropy (membership dispersion), and the ambiguous rate (fraction of samples with maximum membership probability < 0.60).

K = 2 produced coarse partitions with large dominant clusters, consistent with under-segmentation. In contrast, K = 8 resulted in smaller and more fragmented clusters with increased ambiguity. The five-cluster solution maintained balanced cluster sizes and probabilistic interpretability while resolving additional substructure beyond canonical three-class organization.

Method	K	Largest Cluster Fraction	Smallest Cluster Fraction	Cluster Size SD	Ambiguous Rate (p<0.6)	Avg Max Prob	Normalized Entropy
**FCM**	2	0.523	0.477	10.00	0.128	0.785	0.674
**FCM**	5	0.271	0.115	30.03	0.440	0.649	0.599
**FCM**	8	0.158	0.094	10.42	0.537	0.597	0.602
**GMM**	2	0.658	0.342	69.00	0.037	0.936	0.209
**GMM**	5	0.266	0.094	27.17	0.023	0.932	0.113
**GMM**	8	0.229	0.044	24.43	0.030	0.945	0.073

Table S12. Comparison of Fuzzy C-Means (K = 5) clustering performance across embeddings with varying Biological Transition Score (BTS) values.

Metrics include Silhouette score, Calinski–Harabasz (CH) index, Davies–Bouldin (DB) index (lower is better), average maximum membership probability, and proportion of ambiguous assignments (defined as maximum membership probability < 0.6). Ambiguity rates were nontrivial across all embeddings (42.8–57.3%), indicating that mixed-membership structure is not unique to the BTS-selected PCUMAP embedding. PCUMAP was retained due to its combination of strong clustering quality and lower expert-label overlap.

**Method**	**Silhouette**	**CH**	**DB**	**Avg Max Prob**	**Ambiguous rate (p<0.6)**
**PCA**	0.287	263.260	1.082	0.596	0.573
**ISOMAP**	0.313	311.327	1.018	0.617	0.490
**t-SNE**	0.306	329.57	1.093	0.604	0.532
**UMAP**	0.356	440.834	0.979	0.648	0.428
**PCUMAP**	0.351	381.235	0.957	0.643	0.438

Figure S8. Multi-angle views of clustering result from the dendritic spine feature dataset in 3-dimensional PCUMAP embedding space using 3 clustering methods

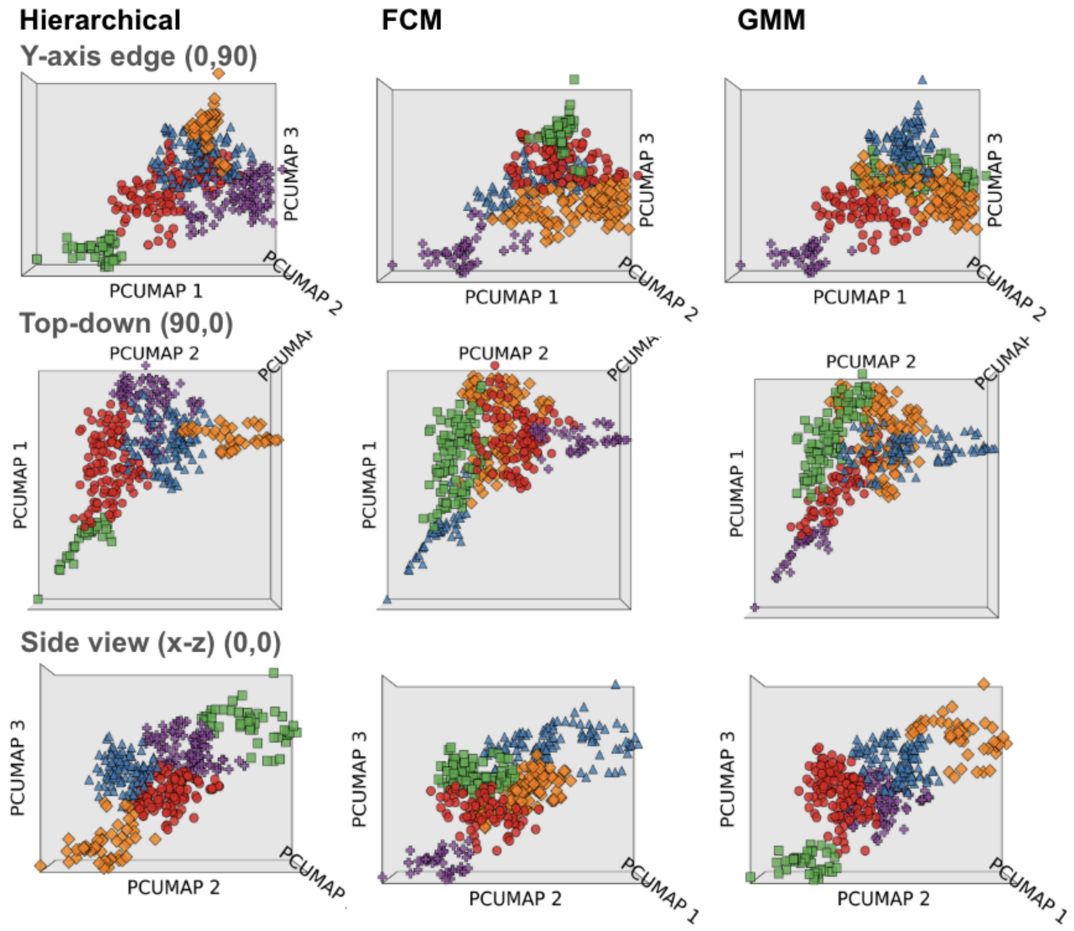

Figure S9. Visualization of cluster membership strength and intermediate structure in the PCUMAP embedding (K=5, FCM).

(Left) Dominant cluster assignment for each spine, where the point size and opacity scale with maximum membership probability, highlighting high-confidence cores and lower-confidence boundary regions. (Right) Normalized entropy of cluster membership (continuum score), where higher values indicate greater ambiguity and overlap across clusters. These plots demonstrate that intermediate regions correspond to gradual transitions in probabilistic membership rather than sharp boundaries, supporting the multivariate structure captured by the framework.

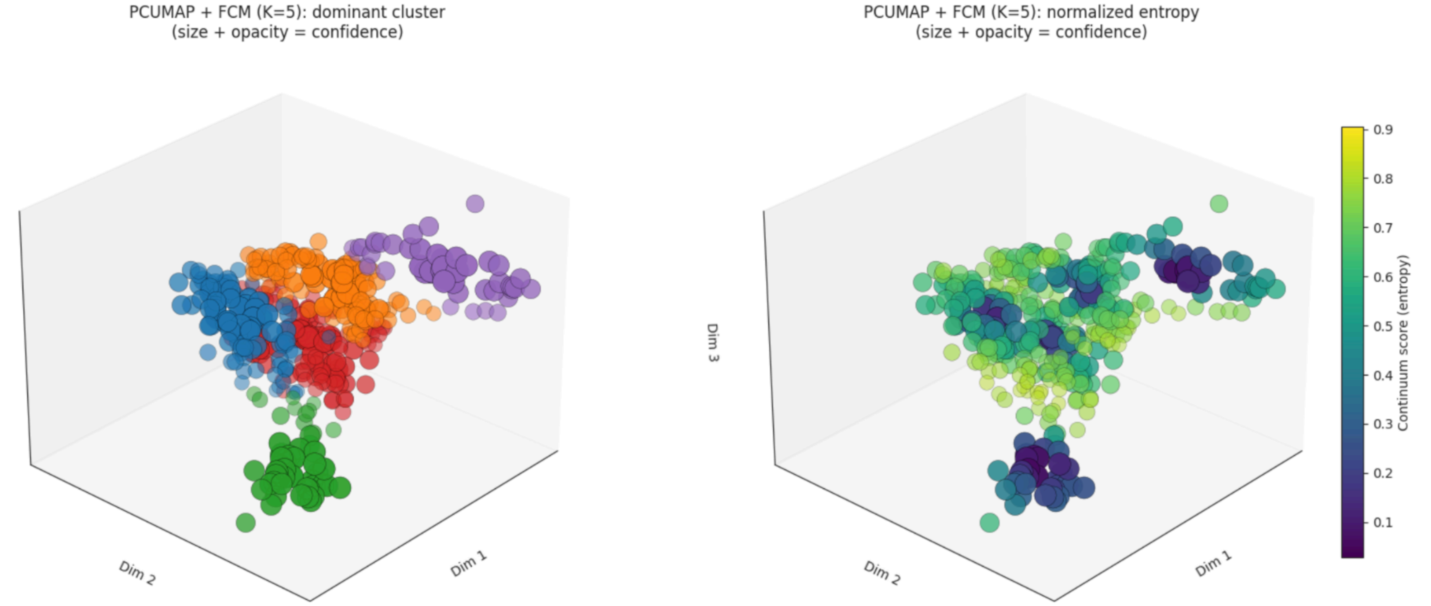

Figure S10. Feature distribution heatmaps for clustering results obtained using fuzzy c-means clustering on PCUMAP projection

In order to understand how each of the features contributes to each cluster, we analyzed the deviation of each feature from its mean value within each cluster. This gives an idea of if certain features are significantly over or underexpressed within a cluster, and also tells us which features contributed the most to the cluster assignments. The features were scaled using StandardScaler to ensure that these values represent proportional shifts.

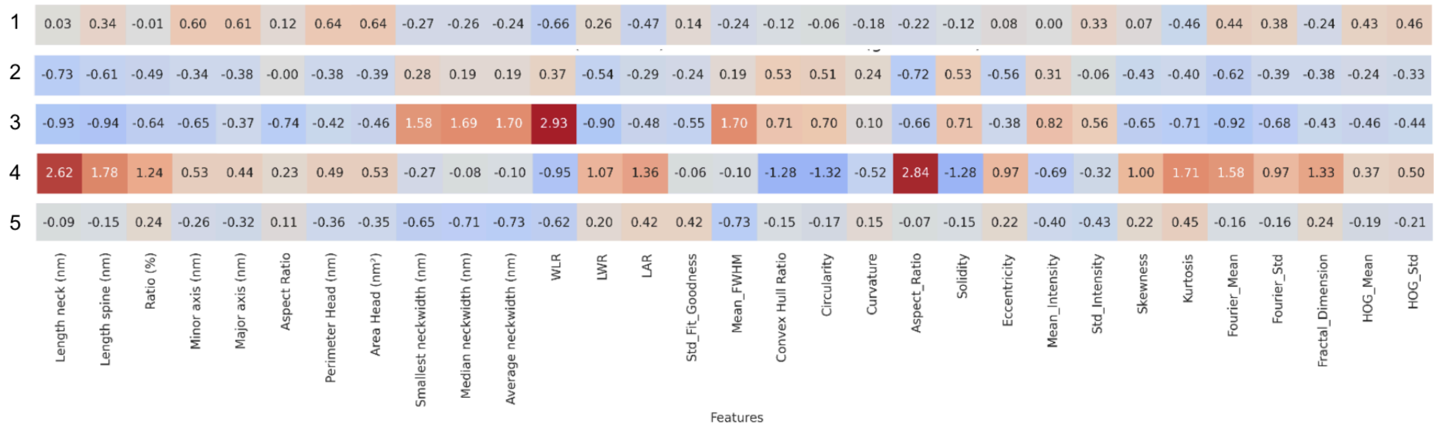

Table S13. Dendritic spines in each cluster

The spines in each cluster are detailed below. The original data can be found here, and spine numbers correspond to their assigned number in the original dataset.

Cluster	Spines in cluster
**1**	291, 293, 294, 209, 26, 217, 289, 267, 285, 269, 236, 237, 238, 388, 263, 246, 83, 390, 392, 254, 256, 287, 298, 152, 200, 69, 316, 177, 319, 321, 44, 45, 180, 325, 349, 53, 342, 158, 50, 156, 340, 326, 135, 41, 31, 127, 194, 302, 306, 199, 65
**2**	172, 423, 422, 421, 420, 425, 335, 424, 396, 427, 187, 428, 430, 432, 433, 434, 336, 437, 438, 439, 440, 266, 451, 224, 426, 341, 395, 407, 414, 405, 410, 415, 416, 400, 399, 404, 82, 129, 409, 403, 406, 99, 398, 417, 419, 408, 397, 411, 94, 92, 378, 402
**3**	89, 100, 86, 91, 103, 85, 104, 386, 90, 385, 93, 48, 79, 2, 5, 9, 11, 12, 16, 18, 444, 443, 23, 435, 42, 43, 105, 51, 413, 72, 75, 76, 80, 383, 252, 381, 170, 320, 174, 175, 179, 185, 312, 311, 310, 303, 197, 211, 214, 216, 169, 218, 284, 223, 228, 276, 275, 274, 273, 271, 232, 233, 234, 241, 242, 245, 286, 382, 324, 163, 106, 108, 111, 113, 376, 115, 370, 119, 123, 125, 126, 130, 131, 134, 167, 356, 143, 144, 145, 346, 345, 344, 148, 151, 155, 157, 161, 162, 332, 330, 353, 138
**4**	1, 361, 360, 136, 359, 358, 357, 355, 351, 350, 146, 343, 150, 337, 334, 333, 327, 323, 259, 322, 365, 122, 95, 391, 389, 102, 387, 384, 380, 107, 110, 112, 377, 114, 372, 371, 369, 120, 121, 366, 171, 317, 176, 455, 280, 279, 278, 277, 268, 239, 240, 243, 247, 248, 249, 250, 251, 255, 257, 258, 221, 290, 215, 295, 178, 181, 315, 183, 184, 314, 309, 189, 393, 190, 301, 300, 299, 297, 296, 206, 207, 208, 304, 394, 132, 260, 445, 74, 21, 442, 68, 67, 19, 37, 38, 412, 40, 60, 59, 58, 64, 449, 28, 78, 453, 14, 4, 3, 87, 8, 454, 88, 452, 7, 6, 10, 450, 13
**5**	367, 339, 261, 262, 264, 348, 329, 328, 373, 305, 368, 282, 292, 283, 281, 446, 447, 448, 362, 379, 363, 270, 313, 364, 354, 352, 318, 375, 308, 265, 272, 226, 456, 164, 165, 166, 52, 168, 49, 46, 173, 124, 39, 182, 36, 186, 35, 34, 33, 139, 55, 57, 61, 128, 81, 140, 141, 137, 142, 77, 133, 147, 191, 73, 149, 70, 154, 66, 63, 159, 160, 62, 71, 192, 188, 195, 98, 222, 193, 225, 227, 118, 229, 230, 117, 20, 116, 17, 231, 15, 101, 235, 244, 220, 24, 22, 25, 32, 196, 30, 198, 219, 202, 204, 205, 201, 97, 27, 210, 253, 212, 29, 213

Table S14. Sensitivity analysis for class imbalance

The original dataset contains an imbalance proportion of spine types, with mushroom spines dominating (n= 288), followed by stubby spines (n= 113) and the lowest number of thin spines (n= 55). For this reason, we wanted to conduct a sensitivity analysis to understand if the results from our clustering algorithm are robust across different dataset scales. We randomly subsampled the original dataset (456 spines) to include an equal proportion of mushroom, stubby, and thin spines, resulting in 144 spines being used for a balanced analysis (48 from each category). The table presents key metrics for the three clustering methods comparing the original (imbalanced) dataset and the balanced dataset.

Across all clustering methods, balancing spine-type representation led to more uniform cluster sizes, as reflected by a consistent decrease in the coefficient of variation (CV) of cluster size. This demonstrates that cluster-size heterogeneity in the original dataset is partially driven by class imbalance. Additionally, balancing does not degrade clustering confidence, with maximum membership probability remaining high and average entropy decreasing, indicating less "fuzzy" cluster assignments in the balanced case. These results suggest that the observed cluster organization reflects intrinsic morphological structure and is not solely driven by spine-type imbalance.

**Clustering Method**	**Cluster sizes (cluster #: # of spines)**		**Coefficient of variation of cluster size (σ/μ)**		**Avg. maximum probability**		**Avg. Entropy**	
	**Original**	**Balanced**	**Original**	**Balanced**	**Original**	**Balanced**	**Original**	**Balanced**
**Hierarchical**	1: 132<br>2: 110<br>3: 39<br>4: 104<br>5: 51	1: 43<br>2: 31<br>3: 18<br>4: 17<br>5: 35	0.460	0.388	–	–	–	–
**FCM**	1: 102<br>2: 53<br>3: 119<br>4: 50<br>5: 112	1: 38<br>2: 29<br>3: 24<br>4: 19<br>5: 34	0.380	0.263	0.972	0.962	5.398	1.075
**GMM**	0: 120<br>1: 100<br>2: 86<br>3: 49<br>4: 81	1: 33<br>2: 24<br>3: 36<br>4: 27<br>5: 24	0.300	0.189	0.930	0.957	0.185	1.116

Figure S11. Distribution of mushroom, stubby, and thin spines across 5 clusters in the original (imbalanced) dataset compared to the balanced dataset.

The confusion matrices detail the proportion of mushroom, stubby, and thin spines contained within each cluster for each clustering method, evaluated on the original dataset (top) and on the balanced subset with equal spine type representation (bottom). Across all three clustering methods, the dominant alignment patterns between clusters and spine types are generally preserved after balancing. Notably, the FCM and GMM results, which incorporate soft assignments, show that clusters associated with thin spines still remain well separated from those dominated by mushroom or stubby spines. Additionally, the balanced analysis doesn't introduce increased mixing between dissimilar spine types (e.g. stubby and thin).

These results show that while equal spine type representation alters the absolute number of spines in each cluster, it still preserves the qualitative structure of cluster–label relationships across clustering algorithms. Note that the "cluster number" is arbitrary, and the takeaway here is instead the proportion of each spine type across each cluster. This analysis supports the conclusion that the observed clustering patterns reflect intrinsic morphological organization captured by our method, rather than just being artifacts of spine-type imbalance.

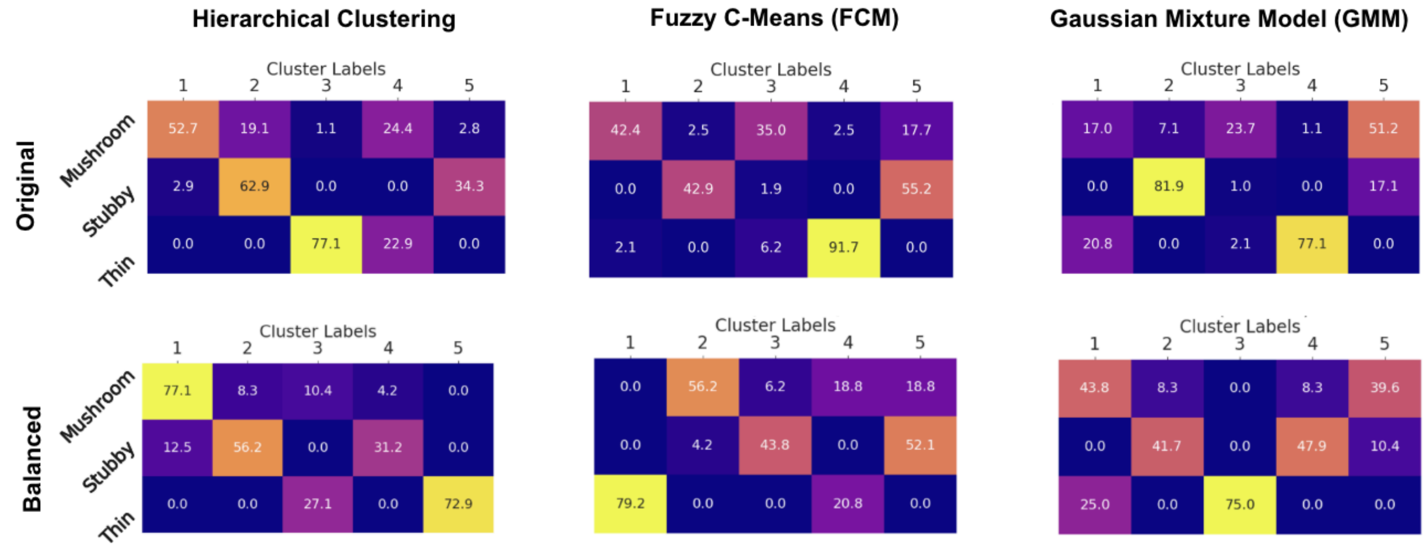

Table S15. Dimensionality reduction results on a secondary dataset (Smirnov et al.)

PCA demonstrated the strongest overall structure preservation (SPS = 0.847), with the highest combined local and global scores. ISOMAP performed comparably but ranked second overall. While t-SNE and UMAP achieved high continuity, they exhibited weaker global preservation. PCUMAP showed reduced global performance in this dataset. These results support selection of PCA as the preferred dimensionality reduction method. It is important to note that this second dataset was lower resolution compared to the one used for the primary analysis, which may have obscured some of the non-linear features and curvatures of the data leading to the linear dimensionality reduction methods performing better at structure preservation.

**Method**	**Trustworthiness**	**Continuity**	**LCMC**	**MRRE**	**Pearson**	**Spearman**	**LS**	**GS**	**SPS**
**PCA**	0.926	0.987	0.724	6.269	0.877	0.861	**0.825**	**0.869**	**0.847**
**ISOMAP**	0.917	0.988	0.684	6.067	0.844	0.828	0.801	0.836	0.818
**t-SNE**	0.909	0.990	0.689	5.451	0.750	0.756	0.799	0.753	0.776
**UMAP**	0.899	0.990	0.686	5.540	0.734	0.723	0.792	0.728	0.760
**PCUMAP**	0.877	0.990	0.621	5.772	0.439	0.775	0.749	0.607	0.678

Table S16. Clustering results on secondary dataset (Smirnov et al.)

On this dataset, FCM achieved the highest silhouette score, highest CH score, a low DB score comparable to hierarchical clustering, the highest average maximum probability, and has the added advantage of providing probabilistic assignments, which is advantageous for modeling dendritic spine morphology. Therefore, FCM was chosen as the preferred clustering method for this dataset.

Method	Silhouette	CH Score	DB Score	Avg Entropy	Avg Sharpness	Avg Max Prob
**FCM**	**0.348**	**138.094**	0.937	4.934	0.093	**0.950**
**GMM**	0.259	95.829	1.148	0.211	**0.869**	0.910
**Hierarchical**	0.311	116.621	**0.927**	—	—	—

Figure S12. Generalization of the clustering framework to the second lower-resolution dataset (Smirnov et al.).

This figure demonstrates the results from applying the proposed decision framework to an independent lower-resolution dataset (Smirnov et al.) to evaluate generalizability across imaging conditions. (A) Structure preservation analysis comparing dimensionality reduction methods using local and global scores, which identified PCA as optimal for this dataset. (B) Silhouette score and distortion curves across cluster numbers (K=2–10), which led to the selection of a five-cluster solution. (C) Three-dimensional PCA embedding with FCM clustering (K=5), which illustrates cluster structure in reduced space. (D) Transition matrix showing cluster adjacency using FCM.

These results indicate that the framework consistently identifies comparable morphometric structure despite differences in resolution and acquisition parameters, supporting its robustness across datasets and imaging conditions.

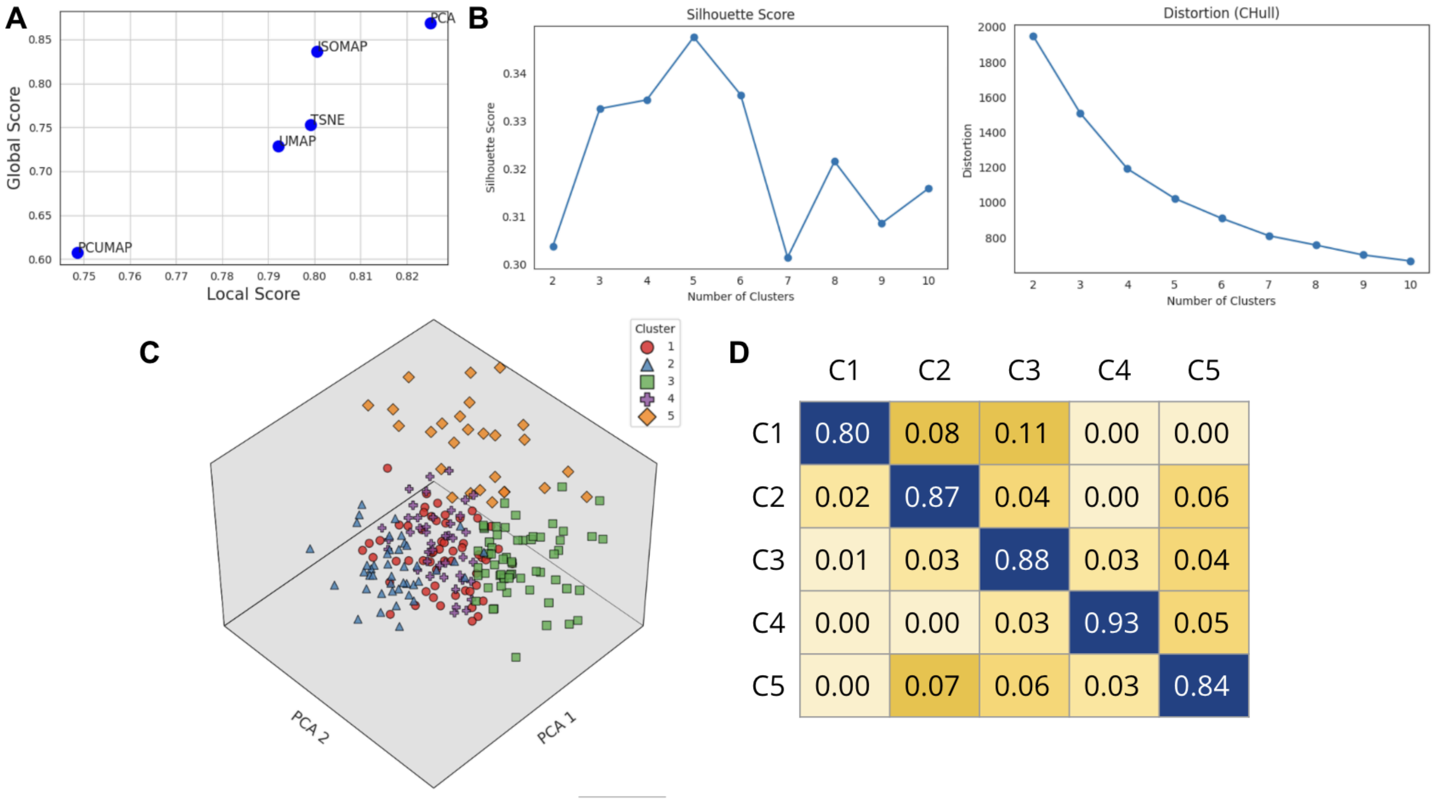

Table S17. Suggested Python package dependencies

pccdr==1.0.1
torchdr==0.2
torch==2.2.2
scikit-learn==1.3.2
fcmeans==1.6.3
umap-learn==0.5.8
numpy==1.26.4
pandas==2.2.2
matplotlib==3.8.3
seaborn==0.13.2

References

1. A. Rodriguez, D. B. Ehlenberger, D. L. Dickstein, P. R. Hof, and S. L. Wearne. Automated three-dimensional detection and shape classification of dendritic spines from fluorescence microscopy images. *PLoS ONE*, 3(4):e1997, 2008.

2. P. Shi, Y. Huang, and J. Hong. Automated three-dimensional reconstruction and morphological analysis of dendritic spines based on semi-supervised learning. *Biomedical optics express*, 5(5):1541–1553, 2014.

3. M. U. Ghani, E. Erdil, S. D. Kanik, A. O. Argunsah, A. F. Hobbiss, I. Israely, D. Unay, T. Tasdizen, and M. Cetin. Dendritic spine shape analysis: A clustering perspective. *Computer Vision – ECCV 2016 Workshops*, 9913, 2016.

4. M. U. Ghani, A. O. Argun¸sah, I. Israely, D. ¨Unay, T. Ta¸sdizen, and M. Cetin. On comparison of manifold learning techniques for dendritic spine classification. *IEEE 13th International Symposium on Biomedical Imaging (ISBI)*, 339–342, 2016.

5. M. U. Ghani, F. Mesadi, S. D. Kanik, A. O. Argunsah, A. Hobbiss, I. Israely, D. Unay, T. Tasdizen, and M. Cetin. Shape and appearance features based dendritic spine classification. *Journal of Neuroscience Methods*, 279:13–21, 2017.

6. G. Bokota, M. Magnowska, T. Kusmierczyk, M. Lukasik, M. Roszkowska, and D. Plewczynski. *Computational approach to dendritic spine taxonomy and shape transition analysis.* Frontiers in Synaptic Neuroscience, 10(140), 2016.

7. S. Luengo-Sanchez, I. Fernand-Espinosa, C. Bielza, R. Benavides-Piccione, P. Larranaga, and J. DeFelipe. 3D morphology-based clustering and simulation of human pyramidal cell dendritic spines. *PLOS Computational Biology*, 14(6), 2018.

8. T. Kashiwagi, S. H. Nishimoto, T. Inagaki, and Y. Hayashi. Computational geometry analysis of dendritic spines by structured illumination microscopy. *Scientific Reports*, 9:12345, 2019.

9. N. Ofer, R. Benavides-Piccione, J. DeFelipe, and R. Yuste. Structural analysis of human and mouse dendritic spines reveals a morphological continuum and differences across ages and species. *eNeuro*, 9(3), 2022.

10. J. Choi, S. Lee, Y. Lee, E. Cho, S. Chang, and W. Jeong. DXplorer: A unified visualization framework for interactive dendritic spine analysis using 3D morphological features. *IEEE Trans Vis Comput Graph*, 29(2):1424–1437, 2022.

11. A.Ö. Argunşah, E. Erdil, M. U. Ghani, Y. Ramiro-Cortés, A. F. Hobbiss, T. Karayannis, M. Çetin, I. Israely and D. Ünay. An interactive time series image analysis software for dendritic spines. *Science Reports,* 12, 12405, 2022.

12. E. Pchitskaya, P. Vasiliev, D. Smirnova, V. Chukanov, and I. Bezprozvanny. SpineTool is an open-source software for analysis of morphology of dendritic spines. *Scientific Reports*, 13(1):10561, 2023.

13. M. H. P. Fernholz, D. A. Guggiana Nilo, T. Bonhoeffer, and A. M. Kist. DeepD3, an open framework for automated quantification of dendritic spines. *PLOS Computational Biology,* 20(2):e1011774, 2024.

14. P. Rousseeuw. Silhouettes: A Graphical Aid to the Interpretation and Validation of Cluster Analysis. *Comput. Appl. Math*., 20, 53-65. Journal of Computational and Applied Mathematics. 20, 53-65, 1987.

15. E. Schubert, M. Koeller, and A. Zimek. Model selection strategies for determining the number of clusters in overlapping clustering. *Algorithmica*, 84(4):1135–1164, 202

16. J. Bourne and K. M. Harris. Do thin spines learn to be mushroom spines that remember? *Current Opinion in Neurobiology*, 17(3):381–386, 2007.

17. K. Runge, C. Cardoso, and A. de Chevigny. Dendritic spine plasticity: Function and mechanisms. *Frontiers in Synaptic Neuroscience*, 12, 2020.
